# Transcranial magnetic stimulation and transcranial direct current stimulation affect explicit but not implicit emotion regulation: a meta-analysis

**DOI:** 10.1186/s12993-023-00217-8

**Published:** 2023-09-19

**Authors:** Xiufu Qiu, Zhenhong He, Xueying Cao, Dandan Zhang

**Affiliations:** 1 Institute of Brain and Psychological Sciences, Sichuan Normal University, Chengdu, 610066 China; 2 School of Psychology, Shenzhen University, Shenzhen, 518060 China

**Keywords:** Transcranial magnetic stimulation, Transcranial direct current stimulation, Emotion regulation, Explicit emotion regulation, Implicit emotion regulation

## Abstract

Emotion regulation (ER) refers to the process through which people influence the occurrence, experience, and expression of emotions. It can be established in an explicit (voluntary) or implicit (automatic) way, both of which are essential for mental and physical well-being. Recent evidence has highlighted the potential of transcranial magnetic stimulation (TMS) and transcranial direct current stimulation (tDCS) targeting the prefrontal cortex (PFC) to modulate ER. However, previous studies have only evaluated the effects of TMS and tDCS on explicit ER, leaving implicit ER relatively unexplored. In this review and meta-analysis, we systematically evaluated the effects of TMS and tDCS over the PFC on the two forms of ER, using both subjective and physiological response as outcome indicators. Twenty-seven studies were included in our study. Both subjective (*Hedges’ g* = − 0.20) and physiological (*Hedges’ g* = − 0.65) results indicated a significant effect of TMS and tDCS targeting PFC on down-regulation of explicit ER, but not implicit ER (*Hedges’ g* = − 0.04). Moreover, moderation analysis indicated that the effect of TMS and tDCS on the down-regulating of subjective experience was moderated by several factors, including stimulation method, target area, target hemisphere, and stimulation timing. Specifically, our results showed that applying TMS or targeting the right PFC, particularly the right ventrolateral prefrontal cortex, or using offline TMS and tDCS produced a larger stimulation effect on ER. In summary, these findings suggest that TMS and tDCS has a positive effect on explicit, but not implicit ER. The distinct TMS and tDCS effect on the two forms of ER help deepen our understanding of TMS and tDCS use and provide valuable insights for the development of tailored TMS and tDCS protocols for explicit and implicit regulation.

**Supplementary Information:**

The online version contains supplementary material available at 10.1186/s12993-023-00217-8.

## Introduction

Emotion regulation (ER) involves individuals modifying their emotional responses to behave appropriately when encountering various social situations, which is essential for maintaining both physical and mental health [1–3]. The cognitive framework of ER suggests that this process occurs either voluntarily (explicit ER) or automatically (implicit ER) [4, 5]. Explicit ER runs with a conscious effort to change emotional responses and requires conscious monitoring, while implicit ER begins automatically and involves the change of emotional responses without monitoring [6]. To assess ER processes/outcomes, studies usually use participants’ subjective experiences (e.g., emotional intensity and valence rating) and physiological indexes (e.g., skin conductance response and pupil dilation) [7–10]. However, alterations in these measures do not consistently mirror each other across distinct ER tasks. Suppression, for example, reduces skin conductance response, leaving emotional intensity unaffected [11, 12], whereas cognitive reappraisal diminishes negative emotional experiences without affecting heart rates [13].

Neuroimaging studies have suggested that explicit and implicit ER both critically involve the prefrontal cortex (PFC) but with specific different PFC subregions. Explicit ER largely recruits the lateral PFC, namely the dorsolateral PFC (DLPFC) and ventrolateral PFC (VLPFC) [14, 15]. In contrast, implicit ER engages more with the medial PFC (MPFC), especially ventral MPFC (VMPFC) [16–18]. ER pursues two different regulation goals, down-regulation (diminishing emotion) and up-regulation (intensifying emotion) [19]. These two goals also are associated with distinct PFC regions: down-regulation is associated more with the right PFC activity, while up-regulation is related more with the left PFC activity [20, 21].

Nevertheless, conclusions deriving from neuroimaging techniques are largely correlational and the causal inference between PFC functioning and ER could not be derived. Non-invasive brain stimulation could temporally modify brain excitability without harm [22], which is a promising tool to investigate such causal relationships. Non-invasive brain stimulation includes transcranial electrical stimulation (tES) and transcranial magnetic stimulation (TMS) techniques. tES applies various current waveforms, including transcranial direct current stimulation (tDCS), transcranial alternating current stimulation (tACS), and transcranial random noise stimulation (tRNS), to the scalp, modulating neuronal states [23]. Among these, tDCS is the most commonly used protocol, delivering low-intensity electrical current (typically 1–2 mA) to the superficial brain regions, thereby modifying cortical excitability [24, 25]. Anodal tDCS enhances cortical activity, while cathodal tDCS exerts the opposite effect [26, 27]. In contrast, TMS applies brief, high-intensity magnetic pulses to the scalp, inducing electric fields that alter neural activity [28]. TMS can be administered as single-pulse TMS (spTMS) or repetitive TMS (rTMS). The effects of rTMS depend on its frequency: low-frequency rTMS (< 1 Hz) or intermittent theta burst stimulation (iTBS) exert inhibitory effects, while high-frequency rTMS (> 5 Hz) or theta burst stimulation (cTBS) can induce excitatory effects [29, 30]. tDCS and TMS are two commonly used brain stimulation methods. Research has demonstrated the efficacy of TMS and tDCS targeting the prefrontal cortex (PFC) in modulating emotion and emotion perception [31–34]. Furthermore, emerging evidence suggests that rTMS and anodal tDCS can enhance PFC activity during emotion regulation, potentially improving emotional regulation abilities [35–39]. However, since individual TMS and tDCS studies on ER varies in stimulation protocols, forms of ER, and measurement methods, evidence is not consistent in all studies and therefore the causal relationship remains inconclusive. Quantifying the TMS and tDCS effects on ER is expected to (1) refine the scope of TMS and tDCS application to maximize its ER-modulating effect, thereby providing an efficient way for people to improve their emotional health and general well-being, and (2) benefit the treatment of ER deficits in psychiatric disorders such as anxiety [40] and depression [41].

TMS and tDCS modulatory effects on ER have been partially summarized in two meta-analysis studies [42, 43]. Specifically, Smith and colleagues demonstrated the efficacy of tDCS in decreasing stress-related emotional reactivity, which may be attributed to the effect of anodal tDCS on ER [42]. This finding indirectly suggests that TMS and tDCS may influence ER process to reduce negative emotional responses. While Zhang and colleagues provided direct evidence that TMS and tDCS reduced negative emotions during down-regulation [43]. However, these studies did not include studies aimed to up-regulate emotion; furthermore, explicit and implicit ER were not differentiated in prior work. Considering that explicit (including both up- and down-regulation) and implicit ER have been demonstrated to be associated with distinct neural representations in PFC [5, 6], it is expected that the effects of TMS and tDCS targeting PFC on explicit and implicit ER may differ. Therefore, a systematic review of the literature on explicit and implicit ER is needed for a comprehensive understanding of the effect of TMS and tDCS on ER. Distinguishing and comparing the TMS and tDCS effects on different forms of ER may also help develop individualized TMS and tDCS protocols targeting various ER deficits.

The current meta-analysis aimed to provide a comprehensive overview of the TMS and tDCS effects on ER, with an assumption that TMS and tDCS differentially modulates the explicit and implicit ER. Considering the potential inconsistency between subjective and physiological on ER, it is necessary to evaluate the effect of TMS and tDCS on ER by using various types of measurements. Thus, in addition to the self-reported emotional feelings [42, 43], we also included physiological responses such as skin conductance response and pupil dilation because they provided objective indices for the effect of TMS and tDCS on ER. Given the high heterogeneity observed in previous meta-analyses [43], we further investigated whether stimulation method and stimulation parameter (e.g., targeted area/hemisphere, stimulation timing, and stimulation duration) moderate the effect of TMS and tDCS on ER. In addition, studies have shown that the cognitive resources recruited during ER differ between general affective pictures and specific affective stimuli during ER. The former, induced by the International Affective Picture System (IAPS) [44], are complex in emotional content and require more cognitive resources, whereas the latter, induced by specific affective stimuli (e.g., pain and memory), exhibit less complex and heterogeneous content, which need relatively fewer cognitive resources [21]. Therefore, it can be speculated that stimuli type may affect the effect of TMS and tDCS on ER.

## Methods

### Literature search

Following the Preferred Reporting Items for Systematic Reviews and Meta-Analyses (PRISMA) guidelines [45], a literature search was conducted by two trained investigators (Xiufu Qiu & Zhenhong He) using the PubMed, Web of Science, and Scopus electronic databases to obtain studies on TMS and tDCS and ER from the earliest publication dates available to March 2023. The combination of keywords “TMS or tDCS or tACS or tRNS” and “ER” was utilized in the search, which was limited to human studies and English-language publications. The detailed search terms can be found in Part 1 of the Additional file 1. Following *Cochrane Handbook for Systematic Reviews of Interventions* [46], reference lists from similar reviews and meta-analyses were also screened for relevant studies [42, 43, 47–51]. This study was pre-registered on the Open Science Forum platform (https://osf.io/87t6s).

### Eligibility criteria

Studies that met the following criteria were included in the meta-analysis: (1) Studies were published in English journals. (2) Participants were healthy human adults aged 18–60 years old. (3) TMS and tDCS was administered over PFC before or during the ER task. The examples of excitatory TMS and tDCS included high-frequency rTMS, iTBS, anodal tDCS, while the examples of inhibitory TMS and tDCS were low-frequency rTMS, cTBS, and cathodal tDCS [23, 52]. (4) The TMS and tDCS protocol included a sham or control condition. For the sham condition of TMS, stimulation was administered through either a sham coil, a tilted coil, or vertex stimulation [53]. For the sham condition of tDCS, a short (usually 30–60 s) application of current was applied at the beginning of tasks and gradually switched off [54]. (5) Studies used explicit ER (i.e., reappraisal, distraction, suppression, distancing, and placebo) or implicit ER tasks (i.e., extinction, reinforcer revaluation, emotional Go/No-Go, emotional Stroop, affect labeling, automatic goal pursuit, and reversal learning) [5]. (6) The effect of TMS and tDCS on ER was measured by the self-reported scores and/or physiological responses, including valence, arousal, and intensity, skin conductance response, fear-potentiated startle, pupil dilation, and facial electromyography.

### Data extraction

Two investigators (Xiufu Qiu & Zhenhong He) independently screened the title, abstract, and full text of the studies. They then extracted all relevant data from the final included articles. Any disagreement was settled by a panel discussion with a third investigator (Dandan Zhang). Specifically, there were 16 disagreements out of 270, which represents a relatively small proportion. Each case was thoroughly reviewed by the three-person group until a consensus was reached. For each article, the following data were extracted: author’s name, publication years, study design, sample size, the stimulation protocol (stimulation method, target area, stimulation parameters, control condition, stimulation timing), affective evoking material (i.e., general affective pictures or specific affective stimuli), task types (e.g., ER task or fear extinction), ER goals (down-regulation or up-regulation), the outcome measures of ER and the results of TMS and tDCS on ER (including the results of subjective experience or physiological response).

For the outcome measures of ER, we extracted the mean (*M*), standard deviation (*SD*), and sample size (*N*) in each condition or group (i.e., active and sham) for further quantitative analyses. The outcome measures were adjusted if necessary. First, in most ER studies, a higher self-reported score indicates more negative emotion. If the study used a reversed scale (i.e., a higher score indicates more positive emotion), the group mean values were normalized to get in line with the typical scale. The equations for the normalization procedure are as follows:

$$ {\text{If}}\,X_{{{\text{med}}}} = \, 0,{\text{ then}}\,X_{{{\text{new}}}} = \, - X_{{{\text{original}}}} $$

$$ {\text{If}}\,X_{{{\text{med}}}} \ne \, 0,{\text{then}}\,X_{{{\text{new}}}} = X_{{{\text{max}}}} - X_{{{\text{original}}}} + {1} $$

where *X*_med_ denotes the median score of the scale, *X*_new_ denotes the normalized mean score, *X*_original_ denotes the original mean score, and *X*_max_ denotes the maximum scale score used in the study. Second, if the study provided the standard error (*SE*) instead of *SD*, *SE* was converted to *SD* through the formulas *SD* = *SE* ×$\sqrt{N}$ [46]. Third, if relevant data were unavailable, we reached out to the corresponding authors. If the data were unable to provide, the data displayed in the figures were identified and extracted by WebPlotDigitizer [55, 56]. We excluded literature only when we cannot obtain data through the above methods.

### Data analysis

All quantitative analyses were performed using Comprehensive Meta-Analysis V3 (CMA, Bio-Englewood, New Jersey, US) [57]. We separately conducted meta-analyses for TMS and tDCS excitability (excitatory and inhibitory TMS and tDCS) and ER measurement (subjective experience and physiological response). For both explicit and implicit ER, we entered the *M*, *SD*, and *N* of the active and sham group into the CMA. Considering that explicit ER involves bidirectional goals (implicit ER typically involves only unidirectional goals, i.e., down-regulation) [20], meta-analyses of down-regulation and up-regulation were also performed separately. In addition, we also performed the above calculates in the no-regulation condition (looking passively at the affective pictures or electric shocks always paired with conditioned stimuli) to ensure the effects of TMS and tDCS were specific to ER processing, rather than general cognitive alteration.

A random-effects model was performed for each meta-analysis due to the methodological diversity among included studies. For each outcome measure, effect size (*Hedges’ g*) was calculated to assess the effect of TMS and tDCS on ER, which can correct the small sample bias [58]. The values of 0.2, 0.5, and 0.8 indicate small, medium, and large effects [59]. Negative values indicate decreased, while positive values indicate increased negative emotional response in active condition compared to sham condition. In general, each study only generated one effect size. If a study reported multiple outcomes from the same participant group, such as different outcome measurements (e.g., valence and arousal) or multiple time points (e.g., early stage and late stage), it may result in multiple effect sizes. However, these multiple effect sizes cannot be independently treated as it would lead to incorrect estimates of the variance for the summary effect size [60]. Therefore, we combined these multiple effect sizes by CMA to obtain an average effect size for each study. For heterogeneity between studies, we used *Cochran’s Q* to identify the presence of heterogeneity and accordingly the *I*^*2*^ was used to measure the magnitude of the heterogeneity, with the values of 25%, 50%, and 75% indicating a small, medium, and large degree of heterogeneity [59]. Funnel plot and Egger’s test were used to evaluate the publication bias if the meta-analysis contains at least 10 different studies [46, 61, 62]. To check the robustness of the results, we conducted a sensitivity analysis using the one-study-removed method with CMA. For all statistical analyses, a *p-value* < 0.05 (two-tailed) was considered significant.

Finally, moderation analysis was used to explore whether *stimulation method* (i.e. tDCS, TMS) and stimulation parameters including *target hemisphere* (left and right PFC), *target area* (regions for explicit ER: lDLPFC, rDLPFC, lVLPFC, and rVLPFC; regions for implicit ER: lVMPFC, rVMPFC, lDLPFC, rDLPFC, lVLPFC, and rVLPFC), *stimulation timing* (online and offline), *stimulation duration* (< 20 min, 20 min, and > 20 min), and *stimuli types* (general affective pictures and specific affective stimuli) influenced the effect of TMS and tDCS on ER. This analysis was only conducted when sufficient data were available (at least 10 studies) [46].

### Risk of bias assessment

Two investigators (Xiufu Qiu & Xueying Cao) independently assessed the risk of bias in each study using the revised Cochrane risk of bias tool (ROB V.2.0) [63]. The following domains were assessed: randomization process, deviations from intended interventions, missing outcome data, measurement of the outcome, and selection of the reported result. The risk of bias for each domain was graded as either low, high, or unclear and then summarized into an overall judgment. A study was regarded as low risk of bias only when all domains were graded as low risk of bias. A study was regarded as unclear risk of bias if one domain was graded as unclear risk of bias and no other domains were graded as high risk of bias. A study was considered as high risk of bias if at least one domain was graded as high risk. Discrepancies between the two investigators were settled by consensus or by a panel discussion with a third investigator.

## Results

### Included literature and study characteristics

**Fig. 1:**
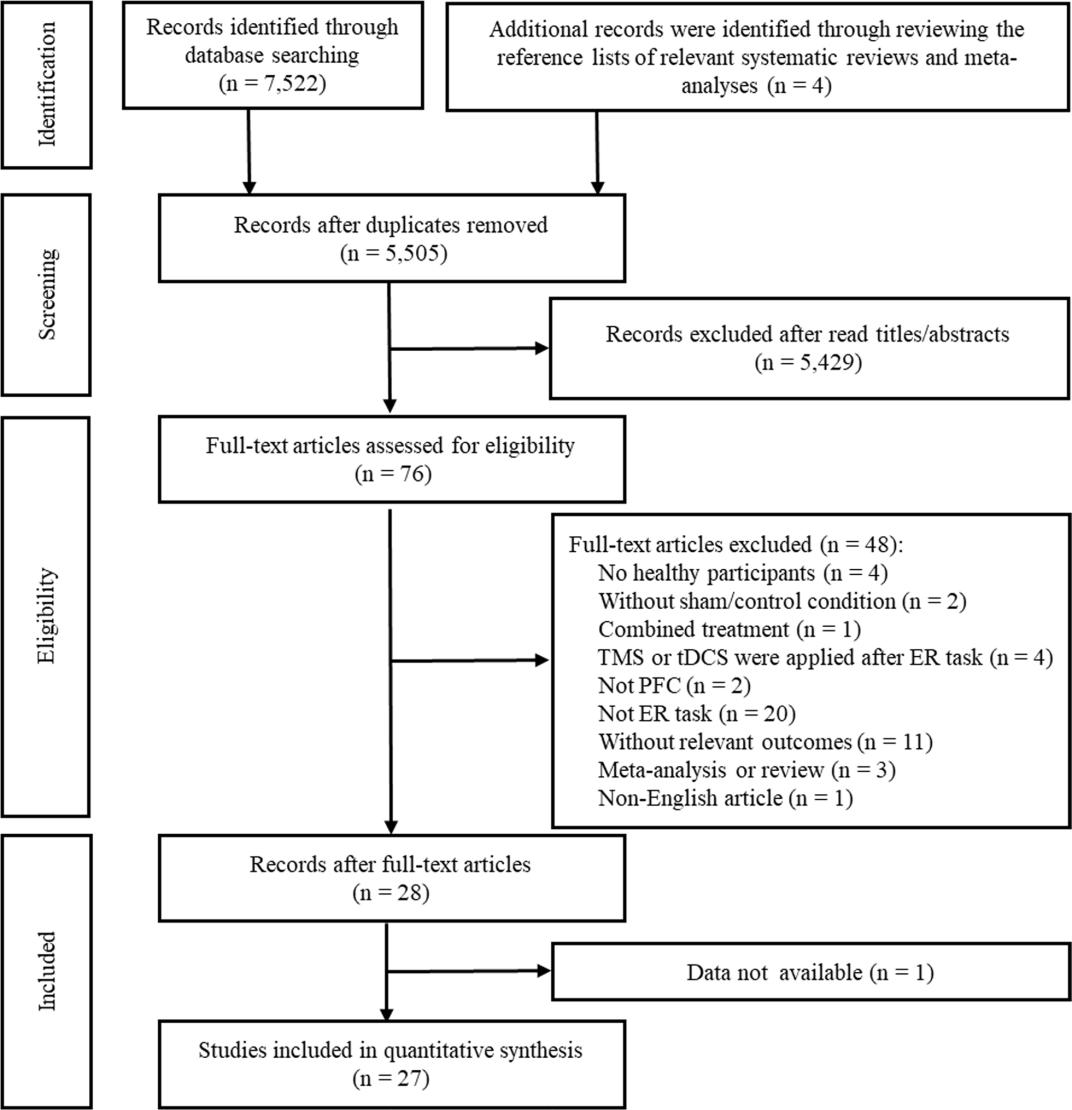
PRISMA flow diagram. PRISMA = Preferred Reporting Items for Systematic Reviews and Meta-Analyses

The systematic search yielded 7522 studies from the database and 4 studies from the reference lists of articles. After removing duplicates, the titles and abstracts of 5505 studies were screened for eligibility. Of these, 76 studies underwent full-text evaluation, and 27 studies that fulfilled the eligibility criteria were included in our review. The literature selection process is visualized in an adapted PRISMA flow diagram (Fig. 1).

Among the 27 studies in our meta-analyses, 2 studies applied both excitatory and inhibitory TMS and tDCS, 21 studies applied excitatory TMS and tDCS, and 4 studies applied inhibitory TMS and tDCS. Therefore, a total of 23 studies were included in the excitatory TMS and tDCS result and 6 studies were included in the inhibitory TMS and tDCS result. Here, we only reported the excitatory TMS and tDCS results in the main text. For the inhibitory TMS and tDCS results, please see the Part 2 in Additional file 1. Among excitatory TMS and tDCS studies, there were 19 explicit and 4 implicit ER studies and included high-frequency rTMS, iTBS, excitatory spTMS, and anodal tDCS. The targeting brain regions included the DLPFC, VLPFC, and VMPFC. For detailed information on study characteristics, see Tables 1, 2.

**Table 1:** Characteristics of excitatory TMS of explicit and implicit ER studies

Author	Design sample size n(active)|n(control)	Coil position (localization method)	Stimulation frequency, quantity, intensity, duration	Control condition	Timing	Stimuli type	Task types	ER goal	Measurement and result
*Explicit ER* (n = 5, k = 7)									
High-frequency rTMS (n = 4, k = 6)									
He et al., 2020a	Between-subjects 30|29	rVLPFC (F8, 10–20)	10 Hz, 1170 pulses, 90% rMT, 15 min	Coil tilted 90◦	Offline	Social exclusion pictures	ERT	down	negative feeling: active < sham
Jansen et al. [75]	Between-subjects 19|17	rDLPFC (F4, neuronavigation)	10 Hz, 3000 pulses, 110% rMT, 5 min	Coil tilted 90◦	Offline	Negative IAPS pictures	ERT	down	negative feeling: active = sham
Li et al. [76] (I)	Between-subjects 40|40^a^	lVLPFC (F7, 10–20)	10 Hz, 800 pulses, 90% rMT, 10 min	Cz	Offline	Negative social feedback	ERT	down	emotional feeling: active = sham
Li et al. [76] (II)	Between-subjects 40|40^a^	rVLPFC (F8, 10–20)	10 Hz, 800 pulses, 90% rMT, 10 min	Cz	Offline	Negative social feedback	ERT	down	emotional feeling: active = sham
Zhao et al. [39] (I)	Between-subjects 30|30^a^	rDLPFC (F4, 10–20)	10 Hz, 624 pulses, 90% rMT, 8 min	Cz	Offline	Social exclusion pictures	ERT	down	negative feeling: active < sham
Zhao et al. [39] (II)	Between-subjects 30|30^a^	rVLPFC (F8, 10–20)	10 Hz, 624 pulses, 90% rMT, 8 min	Cz	Offline	Social exclusion pictures	ERT	down	negative feeling: active < sham
spTMS (n = 1, k = 1)									
Cao et al. [35]	Within-subjects 15|15	lVLPFC (F7, 10–20)	spTMS, 1 pulses, 90% rMT	Cz	Online	Negative IAPS pictures	ERT	down	valence: active < sham arousal: active = sham
*Implicit ER* (n = 2, k = 2)									
iTBS (n = 1, k = 1)									
Deng et al. [79]	Between-subjects 16|19	lDLPFC (F3, 10–20)	30 Hz, 1800 pulses, 80% rMT, 10 min	Cz	Offline	Electrical shock	FET	down	SCR: active = sham
High-frequency rTMS (n = 1, k = 1)									
Guhn et al. [77]	Between-subjects 40|45	rMPFC (NIRS channel 26)	10 Hz, 1560 pulses, 110% rMT, 20 min	Sham coil	Offline	98 db aversive scream	FET	down	arousal: active < sham valence: active = sham FPS, SCR: active = sham

n is the number of studies; k is the number of outcomes

*ER* emotion regulation, *ERT* emotion regulation task, *FET* fear extinction task, *down* down-regulation, *l* left, *r* = right, *VLPFC* ventrolateral prefrontal cortex, *DLPFC* dorsolateral prefrontal cortex, *MPFC* medial prefrontal cortex, *high-frequency rTMS* high-frequency repetitive transcranial magnetic stimulation, *iTBS* intermittent theta burst stimulation, *spTMS* single pulse transcranial magnetic stimulation, *rMT* resting motor threshold, *10–20* 10–20 system for localizing scalp electrodes, *IAPS* international affective picture system, *NIRS* near-infrared spectroscopy, *SCR* skin conductance response, *FPS* fear-potentiated startle

^a^Samples used for multiple experiments within a study

**Table 2:** Characteristics of anodal tDCS of explicit and implicit ER studies

Author	Design sample size n(active)|n(control)	Electrode positions (localization method)	Current intensity, anodal + cathodal size, quantity, duration	Control condition (time of current ramped down)	Timing	Stimuli type	Task types	ER goal	Measurement and result
*Explicit ER* (n = 14, k = 17)									
Chrysikou et al. [65]	Between-subjects 10|10	lDLPFC (anodal, F3; cathodal, F4, 10–20)	1.5 mA, 25 + 25 cm^2^, 20 min	10 s	Online	Negative IAPS pictures	ERT	Down	Negative emotion: active = sham
Clarke et al. [67]	Between-subjects 37|36	lDLPFC (anodal, F3; cathodal, left trapezius, 10–20)	2 mA, 24 + 24 cm^2^, 20 min	30 s	Online	Negative IAPS pictures	ERT	Down	Negative emotion: active = sham
Clarke et al. [67]	Between-subjects 59|57	lDLPFC (anodal, F3; cathodal, left trapezius, 10–20)	2 mA, 24 + 24 cm^2^, 20 min	60 s	Online	Negative IAPS pictures	ERT	Down	Negative emotion: active = sham
Doerig et al. [64]	Between-subjects 50|51	rDLPFC (anodal, rDLPFC; cathodal, vertex, t1-weighted MR)	? mA, 35 + 100 cm^2^, 20 min	30 s	Online	Negative emotional memory	ERT	Down	Valence: active < sham arousal: active = sham
Feeser et al. [36]	Between-subjects 21|21	rDLPFC (anodal, F4; cathodal, Fp1, 10–20)	1.5 mA, 35 + 100 cm^2^, 20 min	30 s	Online	Negative IAPS pictures	ERT	Down	Arousal: active < sham SCR: active < sham
								Up	Arousal: active > sham SCR: active > sham
Fink et al. [73]	Within-subjects 29|29^a^	lDLPFC (anodal, F3; cathodal, Fp2, 10–20)	1 mA, 25 + 25 cm^2^, 20 min	40 s	Online	Disgust pictures	ERT	Down	Disgust: active > sham
Hansenne & Weets [68]	Between-subjects (females only) 20|20	lDLPFC (anodal, F3; cathodal, Fp2, 10–20)	1.5 mA, 9 + 25 cm^2^, 25 min	30 s	Online	Negative IAPS pictures	ERT	Down	Arousal: active = sham
He et al. [38]	Between-subjects 23|21	rVLPFC (anodal, F6; cathodal, Fp1, 10–20)	1.5 mA, 25 + 35 cm^2^, 24 min	30 s	Online	Social exclusion pictures	ERT	Down	Negative emotion: active < sham PD: active < sham
He et al., 2020b	Between-subjects 48|46	rVLPFC (anodal, F6; cathodal, Fp1, 10–20)	2.5 mA, 25 + 25 cm^2^, 34 min	30 s	Online	Social exclusion pictures individual negative pictures	ERT	Down	Negative emotion: active < sham PD: active < sham
Hofhansel et al. [69]	Between-subjects 12|14	rDLPFC (anodal, F4; cathodal, Fp1, 10–20)	1.5 mA, 35 + 100 cm^2^, 20 min	20 s	Offline	Negative IAPS pictures	ERT	Down	Valence: active = sham
								Up	Valence: active = sham
Marques et al. [70] (I)	Between-subjects 30|30^a^	lDLPFC (anodal, F3; cathodal, F4, 10–20)	1.5 mA, 16 + 16 cm^2^, 20 min	30 s	Online	Negative IAPS pictures	ERT	Down	Valence: active = sham arousal: active = sham
								Up	Valence: active = sham arousal: active = sham
Marques et al. [70] (II)	Between-subjects 30|30^a^	rDLPFC (anodal, F4; cathodal, F3, 10–20)	1.5 mA, 16 + 16 cm^2^, 20 min	30 s	Online	Negative IAPS pictures	ERT	Down	Valence: active = sham arousal: active = sham
								Up	Valence: active = sham arousal: active = sham
Marques et al. [70] (III)	Between-subjects 29|30^a^	lVLPFC (anodal, F7; cathodal, F8, 10–20)	1.5 mA, 16 + 16 cm^2^, 20 min	30 s	Online	Negative IAPS pictures	ERT	Down	Valence: active = sham arousal: active = sham
								Up	Valence: active = sham arousal: active = sham
Marques et al. [70] (IV)	Between-subjects 30|30^a^	rVLPFC (anodal, F8; cathodal, F7, 10–20)	1.5 mA, 16 + 16 cm^2^, 20 min	30 s	Online	Negative IAPS pictures	ERT	Down	Valence: active = sham arousal: active = sham
								Up	Valence: active = sham arousal: active = sham
Tu et al. [72]	Between-subjects 27|27	rDLPFC (anodal, F4; cathodal, FP1, 10–20)	2 mA, 16 + 16 cm^2^, 20 min	15 s	Online	Heat stimuli	PNT	Down	Pain rating: active = sham
								Up	Pain rating: active < sham
Van Dam & Chrysikou, [71]	Between-subjects 11|7	lDLPFC (anodal, F3; cathodal, contralateral mastoid, 10–20)	1.5 mA, 25 + 25 cm^2^, 20 min	10 s	Online	Negative IAPS pictures	ERT	Down	Negative emotion: active = sham
Vieira et al. [74] (I)	Between-subjects 11|11^a^	lVLPFC (anodal, F7; cathodal, Fp2, 10–20)	1 mA, 9 + 25 cm^2^, 20 min	30 s	Online	Negative IAPS pictures	ERT	Down	Arousal: active > sham
								Up	Arousal: active = sham
*Implicit ER* (n = 3, k = 4)									
Dittert et al. [78] (I)	Between-subjects 37|26	lVMPFC (anodal, beneath F7; cathodal, beneath F8, 10–20)	1.5 mA, 16 + 16 cm^2^, 20 min	10 s	Offline	95-db loud female scream fearful face	FET	Down	SCR (early extinction): active < sham SCR (late extinction): active = sham
Dittert et al. [78] (II)	Between-subjects 40|27	rVMPFC (anodal, beneath F8; cathodal, beneath F7, 10–20)	1.5 mA, 16 + 16 cm^2^, 20 min	10 s	Offline	95-db loud female scream fearful face	FET	Down	SCR (early extinction): active < sham SCR (late extinction): active = sham
Van’t Wout et al. [80]	Within-subjects 44|44^a^	lVMPFC (anodal, AF3; cathodal, contralateral mastoid, 10–20)	2 mA, 6.96 + 6.96 cm^2^, 20 min	30 s	Offline	Electrical shock	FET	Down	SCR (early extinction): active = sham SCR (late extinction): active < sham

The n is the number of studies; k is the number of outcomes

*ER* emotion regulation, *ERT* emotion regulation task, *FET* fear extinction task, *PNT* placebo nocebo task, *down* down-regulation, *up* up-regulation, *l* left, *r* right, *VLPFC* ventrolateral prefrontal cortex, *DLPFC* dorsolateral prefrontal cortex, *VMPFC* ventral medial, *Fp1* left supraorbital region, *Fp2* right supraorbital region, anodal *tDCS* anodal transcranial direct current stimulation, *10–20* 10–20 system for localizing scalp electrodes, *IAPS* International Affective Picture System, *SCR* skin conductance response, *PD* pupil dilation

^a^Samples used for multiple experiments within a study

### Risk of bias

**Fig. 2:**
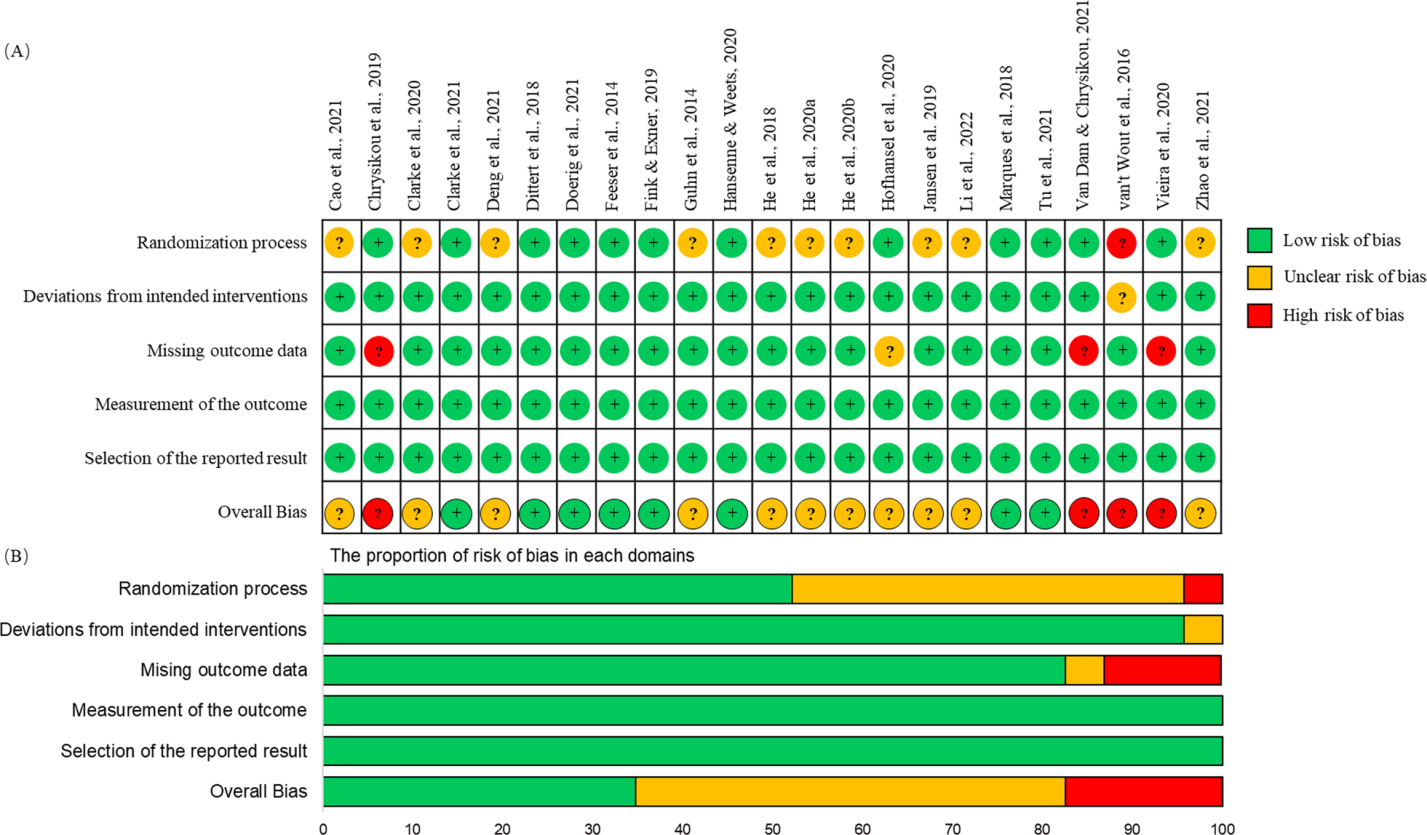
Risk of bias summary of all included studies (n = 23). **A** Methodological quality assessment of each study at 5 domains was illustrated. **B** Risk of bias graph

A summary of the risk of bias assessment of all included studies is illustrated in Fig. 2. Overall, 8 studies (34.8%) were considered as low risk of bias and 11 studies (47.8%) were assessed as unclear risk of bias mainly due to lack of random sequence generation and allocation concealment (43.5%), while 4 studies (17.4%) showed a high risk of bias because of missing outcome data (13%).

### The effect of excitatory TMS and tDCS on explicit ER

#### Subjective experience of down-regulation

The 19 excitatory TMS and tDCS studies reported 24 outcomes on subjective experience of down-regulation and included 14 anodal tDCS studies, 1 spTMS study, and 4 high-frequency rTMS studies. They included 1161 participants, of which 44 underwent both active and sham stimulation, 656 underwent active stimulation and 515 underwent sham stimulation.

A total of 14 studies assessed the effect of anodal tDCS on down-regulation. Four studies targeting the rDLPFC [36, 64] or rVLPFC [38, 41] found a significant anodal tDCS-induced decrease in negative emotional reactivity during down-regulation. The other eight studies primarily stimulated the lDLPFC and didn’t find such an effect [65–72]. Two studies targeting the lDLPFC [73] or lVLPFC [74] found an increase in experienced disgust or arousal after watching negative pictures.

A total of 5 studies assessed the effect of TMS on down-regulation. Two high-frequency rTMS studies targeting the rVLPFC or rDLPFC found a decrease in perceived negative emotion during social pain image presentation [37, 39]. One excitatory spTMS study targeting the lVLPFC also found decreased emotional valence during down-regulating negative pictures [35]. In contrast, two high-frequency rTMS studies observed no effect during down-regulating negative social feedback or negative image after stimulating the VLPFC or DLPFC [75, 76].

**Fig. 3:**
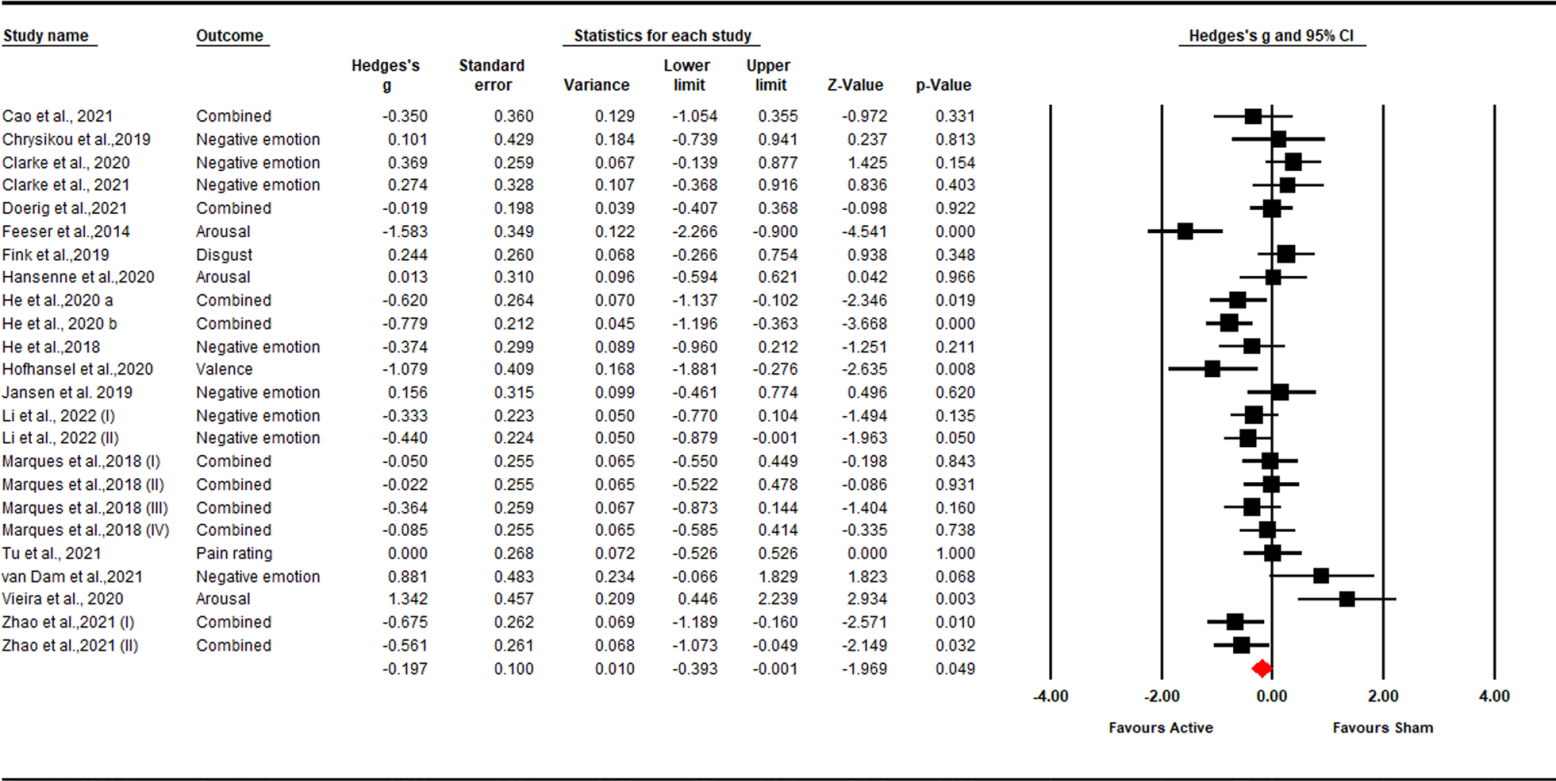
Forest plot for the summary effect size on the effect of excitatory TMS and tDCS on the subjective experience of down-regulation. Combined: Studies with multiple outcomes (e.g., valence and arousal) within a study were combined into an averaged data with CMA, which can prevent an improper estimate of the precision of the summary effect

**Fig. 4:**
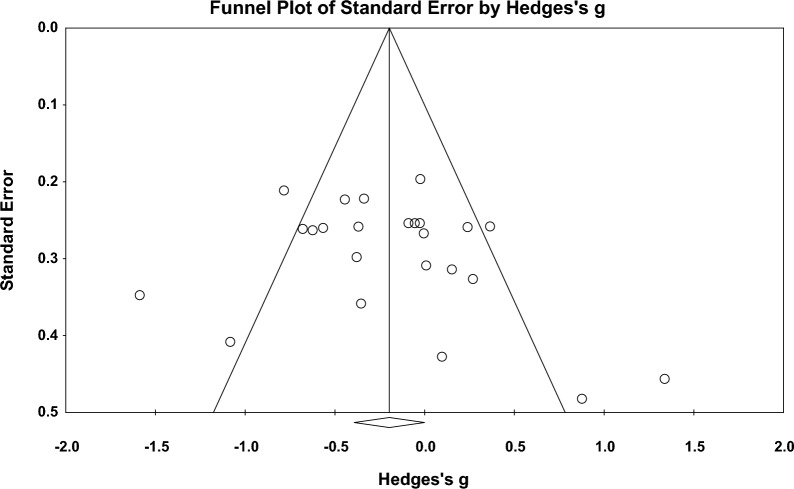
Funnel plot for the excitatory TMS and tDCS effect on the subjective experience of down-regulation, which shows no publication bias; the Egger’s test is non-significant (p = 0.265)

The full random-effects model showed a significant excitatory stimulation effect on subjective experience of down-regulation (*Hedges’ g* = − 0.20; *Z*-value = − 1.97; 95% CI = [− 0.39, 0.00]; *p* = 0.049; Fig. 3), which indicates that excitatory stimulation decreased the negative emotional experience during down-regulation compared to the sham condition. Sensitivity analysis showed that the result was robust (see in the Additional file 1: Fig S5). Moderate heterogeneity was observed (*Q* = 68.61, *p* < 0.001; *I*^*2*^ = 66.47%). Publication bias was not observed through the visual inspection of the funnel plot (Fig. 4) or Egger’s test (*t* = 1.20; *p* = 0.284). The moderation analysis showed that the effect of excitatory stimulation was significantly moderated by the *stimulation method* (*Q* = 4.02, *p* = 0.045)*, target hemisphere* (*Q* = 9.17, *p* = 0.002), *target area* (*Q* = 22.26, *p* = 0.000), and *stimulation timing* (*Q* = 9.95, *p* = 0.019). Further analysis of these moderating variables is as follows: for *stimulation method*, effect sizes of TMS studies were significantly larger than anodal tDCS studies (TMS: *g* = − 0.43, 95% CI [− 0.62, − 0.23]; *p* = 0.000; anodal tDCS: *g* = − 0.10, 95% CI = [− 0.36, 0.17]; *p* = 0.473). For *target hemisphere*, effect sizes of right PFC studies were significantly larger than left PFC studies (right PFC: *g* = − 0.44, 95% CI [− 0.67, − 0.20]; *p* = 0.000; left PFC: *g* = 0.11, 95% CI [− 0.15, 0.37]; *p* = 0.405). In terms of *target area,* effect sizes of rVLPFC studies were significantly larger than lDLPFC, lVLPFC, and rDLPFC studies (rVLPFC: *g* = 0.075, 95% CI [− 0.70, − 0.30]; *p* = 0.000; lDLPFC: *g* = 0.21, 95% CI [− 0.02, 0.43]; *p* = 0.473; lVLPFC: *g* = -0.02, 95% CI [− 0.63, 0.60]; *p* = 0.961; rDLPFC: *g* = -0.42, 95% CI [− 0.85, 0.02]; *p* = 0.059). For *stimulation timing*, effect sizes of offline studies were significantly larger than online studies (offline: *g* = − 0.52, 95% CI [− 0.75, − 0.28]; *p* = 0.000; online: *g* = − 0.05, 95% CI [− 0.29, 0.18]; *p* = 0.405). We also compared brain subregions within *left* and *right PFC* separately. Within the *right PFC*,no significant difference was found between the rDLPFC and rVLPFC (*Q* = 0.12, *p* = 0.734). Similarly, within the *left PFC*, no significant difference was found between the lDLPFC and rVLPFC (*Q* = 0.44, *p* = 0.506). No other significant moderators were found (*p* > 0.05). Details of the moderation analysis were shown in Table 3.

**Table 3:** Moderation analysis results for the subjective experience of down-regulation

	*k*	*Hedges’ g* (95% CI)	*p*^*a*^	*Q*	*df*	*p*^*b*^
*Stimulation method*				4.02	1	**0.045**
tDCS	17	− 0.10, (− 0.36, 0.17)	0.473			
TMS	7	− 0.43, (− 0.62, − 0.23)	** < 0.001**			
*Target hemisphere*				9.17	1	**0.002**
Left PFC	11	0.11, (− 0.15, 0.37)	0.405			
Right PFC	13	− 0.44, (− 0.67, − 0.20)	** < 0.001**			
*Left PFC*				0.44	1	0.506
lDLPFC	7	0.21, (− 0.02, 0.43)	0.075			
lVLPFC	4	− 0.16, (− 0.63, 0.60)	0.961			
*Right PFC*				0.12	1	0.734
rDLPFC	7	− 0.42, (− 0.85, 0.02)	0.059			
rVLPFC	6	− 0.50, (− 0.70, − 0.30)	< 0.001			
*Target area*				22.26	3	**0.000**
lDLPFC	7	0.21, (− 0.02, 0.43)	0.075			
lVLPFC	4	− 0.16, (− 0.63, 0.60)	0.961			
rDLPFC	7	− 0.42, (− 0.85, 0.02)	0.059			
rVLPFC	6	− 0.50, (− 0.70, − 0.30)	** < 0.001**			
*Stimulation timing*				7.32	1	**0.007**
Offline	7	− 0.52, (− 0.85, − 0.20)	** < 0.001**			
Online	17	− 0.06, (− 0.28, 0.16)	0.604			
*Stimuli type*				2.50	1	0.144
General affective pictures	14	− 0.06, (-0.37, 0.26)	0.743			
Specific affective stimuli	10	− 0.36, (-0.57, − 0.15)	0.001			
*Stimulation duration*				5.94	2	0.060
< 20 min	6	− 0.47, (− 0.71, − 0.24)	0.000			
> 20 min	3	− 0.35, (− 0.71, 0.01)	0.054			
20 min	14	− 0.02, (− 0.31, 0.27)	0.873			

Significant *p* values were highlighted in bold

*p*^a^ the p value for effect size (*Hedges' g*), *p*^b^ the *p* value for heterogeneity test (*Cochran’s Q*), *CI* confidence interval, *df* degree of freedom, *Q* Cochran’s Q, assess the presence of heterogeneity, TMS repetitive transcranial magnetic stimulation, tDCS transcranial direct current stimulation, l left, r right, PFC prefrontal cortex, VLPFC ventrolateral prefrontal cortex, DLPFC dorsolateral prefrontal cortex

#### Physiological response of down-regulation

We identified 3 anodal tDCS studies that reported 3 outcomes of the physiological response of down-regulation. These studies included 180 participants, of which 92 underwent active stimulation and 88 underwent sham stimulation. These studies targeting the rDLPFC [36] or rVLPFC [38, 41] found a significant decrease in skin conductance response or pupil dilation to negative stimulation after anodal tDCS.

**Fig. 5:**
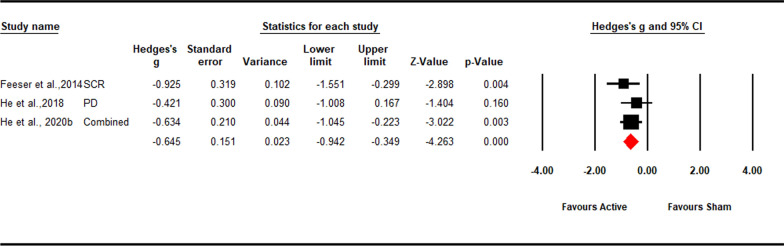
Forest plot for the summary effect size on the effect of excitatory TMS and tDCS on the physiological response of down-regulation. Combined: Studies with multiple physiological outcomes (e.g. individual negative and social negative image) within a study were combined into an averaged data with CMA, which can prevent an improper estimate of the precision of the summary effect; *SCR*  skin conductance response, *PD* pupil dilation

Excitatory stimulation effect on physiological response of down-regulation was significant (*Hedges’ g* = -0.65, *Z*-value = − 4.26, 95% CI [− 0.94, − 0.35], *p* < 0.001; Fig. 5). Sensitivity analysis showed that the result was robust (see in the Additional file 1: Fig S6). Low heterogeneity was observed (*Q* = 1.33; *p* = 0.514; *I*^*2*^ = 0.00%). Due to the small sample size, moderation analysis could not be performed.

#### Subjective experience of up-regulation

We identified 5 anodal tDCS studies that reported 8 outcomes on the subjective experience of up-regulation. These studies included 263 participants, of which 190 underwent active stimulation and 133 underwent sham stimulation. Only one study targeting rDLPFC found an increase in subjective emotional arousal following anodal tDCS [36]. Three studies found no effect [69, 70, 74]. The other study found that anodal tDCS inhibited up-regulation during the nocebo hyperalgesia task [72].

**Fig. 6:**
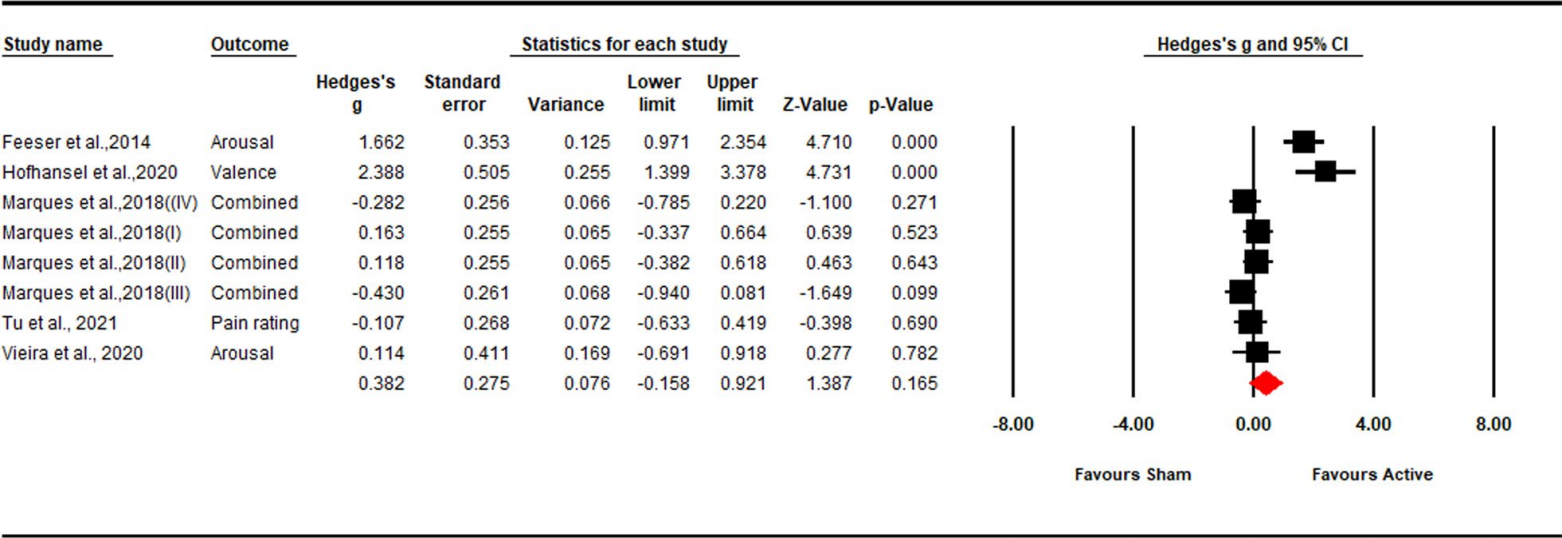
Forest plot for the summary effect size on the effect of excitatory TMS and tDCS on the subjective experience of upregulation. Combined: Studies with multiple outcomes (e.g. valence and arousal) within a study were combined into an averaged data with CMA, which can prevent an improper estimate of the precision of the summary effect

Excitatory stimulation effect on subjective experience of up-regulation was not significant (*Hedges’ g* = 0.38, *Z*-value = 1.39, 95% CI [− 0.15, 0.92], *p* = 0.165; Fig. 6). Sensitivity analysis showed that the result was robust (see in the Additional file 1: Fig S7). High heterogeneity was observed (*Q* = 46.71; *p* < 0.001; *I*^*2*^ = 85.01%). Due to the small sample size, moderation analysis could not be performed.

#### Physiological response of up-regulation

We identified one study examining the effect of excitatory stimulation on the physiological response of up-regulation [36], which found an increase in SCR.

### The effect of excitatory TMS and tDCS on implicit ER

#### Subjective experience

We identified one study examining the effect of excitatory stimulation on the subjective experience of implicit ER [77], which found no effect.

#### Physiological response

We identified 4 studies examining the effect of excitatory stimulation on physiological arousal of implicit ER. These studies included 271 participants, of which 44 underwent both active and sham stimulation, 133 underwent active stimulation and 117 underwent sham stimulation. One tDCS study targeting VMPFC found a decrease in the SCR to an aversive stimulus [78]. However, one tDCS study and two high-frequency rTMS studies found no effect [77, 79, 80].

**Fig. 7:**
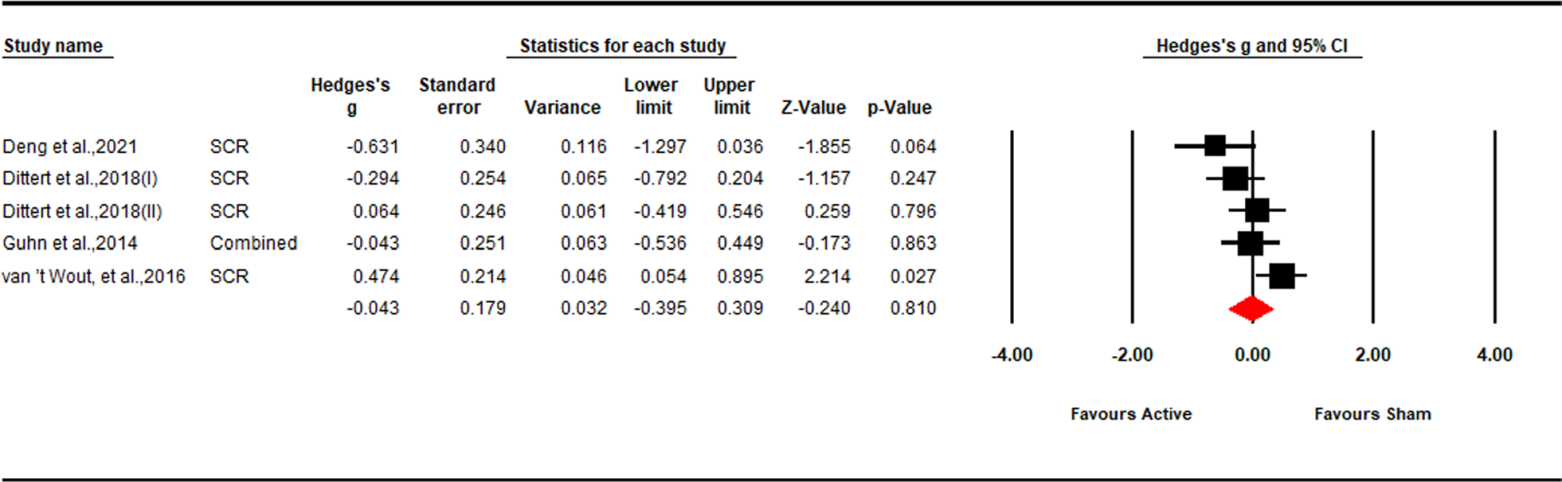
Forest plot for the summary effect size on the effect of excitatory TMS and tDCS on the physiological response of implicit ER. Combined: Studies with multiple outcomes (e.g. SCR and FPS) within a study were combined into averaged data with CMA, which can prevent an improper estimate of the precision of the summary effect; *SCR* skin conductance response, *FPS* fear-potentiated startle

The full random effects model showed no excitatory stimulation effect (*Hedges’ g* = − 0.04, *Z*-value = − 0.24, 95% CI [− 0.40, 0.30], *p* = 0.810; Fig. 7). Sensitivity analysis showed that the result is robust (see in the Additional file 1: Fig S8). Moderate heterogeneity was observed (*Q* = 9.77, *p* = 0.044; *I*^*2*^ = 59.06%). Due to the small sample size, moderation analysis could not be performed.

### The effect of TMS and tDCS on no-regulation condition

**Fig. 8:**
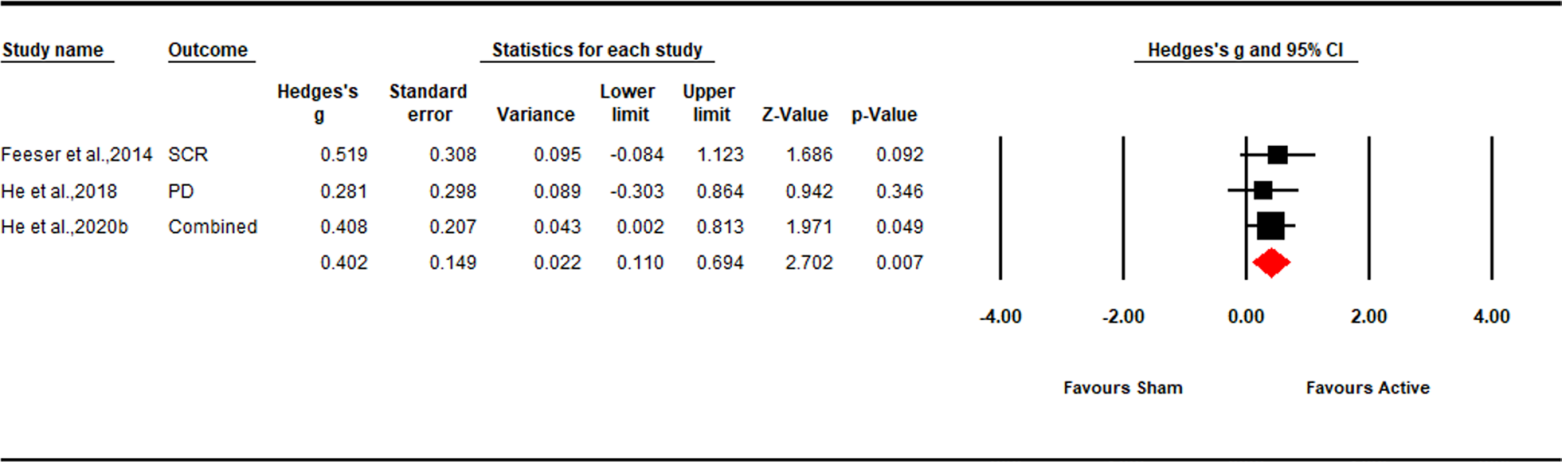
Forest plot for the summary effect size on the effect of excitatory TMS and tDCS on the physiological response of down-regulation advantage. Combined: Studies with multiple outcomes (e.g. individual negative and social negative image) within a study were combined into an averaged data with CMA, which can prevent an improper estimate of the precision of the summary effect; *SCR* skin conductance response, *PD*  pupil dilation

There was no significant TMS and tDCS effect on the self-reported and physiological results (see part 3 in Additional file 1). However, we found a decrease on the physiological response in the control condition of down-regulation after excitatory TMS and tDCS (*Hedges’ g* = − 0.63, *Z*-value = − 0.93, 95% CI [− 0.93, − 0.34], *p* < 0.001). As a result, we performed a meta-analysis of the down-regulation advantage (the differential rating between no-regulation and down-regulation condition) to further interpret the effect of TMS and tDCS on physiological response of down-regulation (see also [41]). The results showed a significant TMS and tDCS effect on physiological response of down-regulation advantage (*Hedges’ g* = 0.40, *Z*-value = 2.70, 95% CI [0.11, 0.49], *p* = 0.007; Fig. 8), which is consistent with the result of physiological response of down-regulation.

### Comparative analysis of types of ER, types of measurement, and ER goals

First, moderation analysis was performed on *types of ER* as a moderator variable during explicit ER (including only down-regulation). A significant difference was found when comparing explicit and implicit ER in physiological response (*Q* = 5.33, *p* = 0.021): there was a positive effect of TMS and tDCS on explicit ER (*k* = 3, *g* = − 0.65, 95% CI [− 0.94, − 0.39], *p* < 0.001) compared with implicit ER (*k* = 4, *g* = − 0.03, 95% CI [− 0.46, 0.39], *p* = 0.881). Due to the small sample size for implicit ER (*k* = 1), the comparison using subjective experience could not be performed.

Moderation analysis was performed on *types of ER* as a moderator variable during explicit ER (including both down- and up-regulation). A significant difference was found when comparing explicit and implicit ER in physiological response (*Q* = 5.17, *p* = 0.023): there was a positive effect of TMS and tDCS on explicit ER (*k* = 3, *g* = − 0.64, 95% CI [− 0.93, − 0.34], *p* < 0.001) compared with implicit ER (*k* = 4, *g* = − 0.03, 95% CI [− 0.47, 0.40], *p* = 0.880). Due to the small sample size for implicit ER (*k* = 1), the comparison using subjective experience could not be performed.

Second, moderation analysis was performed on *ER goals* as a moderator variable during explicit ER (because implicit ER does not have an ER goal). There was no significant difference when comparing down- (*k* = 24, *g* = − 0.20, 95% CI [− 0.39, 0.00], *p* = 0.048) and up-regulation goals (*k* = 8, *g* = 0.38, 95% CI [− 0.16, 0.92], *p* = 0.165) in subjective experience (*Q* = 0.40, *P* = 0.528). Due to the small sample size for up-regulation (*k* = 1), the comparison using physiological response could not be performed.

Third, moderation analysis was performed on *Types of measurement* as a moderator variable explicit ER (including only down-regulation). We observed a significant difference when comparing subjective and physiological responses (*Q* = 6.14, *p* = 0.013): TMS and tDCS can effectively modulate physiological response (*k* = 3, *g* = − 0.65, 95% CI [− 0.94, − 0.35]; *p* < 0.001) of down-regulation compared with subjective experience (*k* = 19, *g* = − 0.17; 95% CI [− 0.42, 0.08]; *p* = 0.189).

Moderation analysis was performed on *Types of measurement* as a moderator variable during explicit ER (including both down- and up-regulation). We also observed a significant difference when comparing subjective and physiological responses (*Q* = 4.78, *p* = 0.030): TMS and tDCS can effectively modulate physiological response (*k* = 3, *g* = − 0.64, 95% CI [− 0.93, − 0.34]; *p* < 0.001) of down-regulation compared with subjective experience (*k* = 19, *g* = − 0.21; 95% CI [− 0.46, 0.04]; *p* = 0.106).

## Discussion

The meta-analysis investigated the potential effect of excitatory TMS and tDCS on ER. Both subjective experience and physiological indexes indicated a significant TMS and tDCS effect on explicit ER (down-regulation), but not implicit ER. In addition, the identified TMS and tDCS effect on down-regulation during explicit ER were moderated by factors including *stimulation method*, *target area/hemisphere*, and *stimulation timing*.

### Effects of TMS and tDCS on explicit and implicit ER

For down-regulation of explicit ER, meta-analysis indicated that TMS and tDCS had a positive effect on subjective experience outcomes (*Hedges’ g* = − 0.20). Such an effect was also identified in a previous meta-analysis, as evidenced by a prominent TMS and tDCS -evoked decrease in self-reported negative emotion [43]. Notably, we also observed a similar and stronger TMS and tDCS effect on physiological outcomes (*Hedges’ g* = − 0.65), which further validates the effectiveness of TMS and tDCS on down-regulation in a more objective way. However, it is important to note that a direct comparison between subjective and physiological outcomes revealed a significant difference (*p* = 0.013), indicating that TMS and tDCS can effectively modulate physiological outcomes of down-regulation but may have limited impact on subjective experience outcomes. One possible explanation for this discrepancy is that the result of subjective experience showed a high heterogeneity in methodology and was modulated by factors stimulation method, target area, target hemisphere, and stimulation timing (see moderation analysis result). Although the results of this study suggest that subjective emotional experiences and physiological responses are incongruent, it is important to note, as described in the Introduction, that these two indicators do not represent identical meanings. Readers should be mindful of this when interpreting the findings. For up-regulation studies, no significant TMS and tDCS effect was found. Evidence demonstrated that up-regulation was associated with more left-lateralized PFC activity, while down-regulation was linked to more right-lateralized PFC activity [20, 21, 81, 82]. In our meta-analysis, most of the up-regulation research has focused on rDLPFC [36, 69, 70, 72], because the primary purpose of these research was not to specifically investigate the effect of TMS and tDCS on up-regulation but rather incidental. Therefore, further research is needed to explore the potential effect of TMS and tDCS targeting the left PFC on up-regulation. In addition, the direct comparison between down- and up-regulation goals of explicit ER suggested no significant differences. Overall, these findings highlight the potential benefits of TMS and tDCS -PFC in improving physiological response of down-regulation, while the evidence for its positive effects in subjective experience of explicit ER (down- and up-regulation) is limited.

Evidence from functional imaging studies has revealed that explicit and implicit ER rely on distinct neurocircuits. During explicit ER, the DLPFC and VLPFC modulate the activity in the lateral amygdala subdivision to block the perceptual and semantic inputs [83], whereas during implicit ER, the VMPFC suppresses the activity in the central amygdala subdivision to inhibit the expression or output of emotional response [16, 84]. By perturbing the two prefrontal-subcortical circuits, it is expected that TMS and tDCS affects both forms of ER, but maybe to a different extent. However, our result did not show a significant effect of TMS and tDCS on implicit ER, and the direct comparison of explicit and implicit ER also supported it. One possible explanation is that the effect of TMS and tDCS is easy to reach superficial regions like LPFC, while accessing deeper cortical areas like VMPFC may pose a challenge [85, 86]. Although researchers have utilized the functional connectivity of DLPFC-VMPFC to indirectly modulate the activity of VMPFC by targeting DLPFC [79, 87], functional imaging study had failed to detect a direct connection between the target region and VMPFC [88]. Therefore, standard TMS and tDCS may not effectively stimulate VMPFC to regulate implicit ER. Novel brain stimulation methods, such as high-definition tDCS (HD-tDCS) and deep TMS, enable targeting deep cortical structures, including VMPFC [89, 90]. Evidence demonstrated that HD-tDCS and deep TMS targeting the VMPFC could effectively modulate aggressive responses, social feedback, and theory of mind [91–93]. Future research should assess the potential effect of HD-tDCS and deep TMS targeting VMPFC for implicit ER.

Overall, our research distinguishes the different effects of TMS and tDCS on explicit and implicit ER, which indicates that future research needs to tailor TMS and tDCS protocols for explicit and implicit ER.

### Factors moderating the effect of excitatory TMS and tDCS on subjective experience of down-regulation

Owing to a large number (n = 19) of the eligible studies, we performed moderation analyses for the subjective experience of down-regulation. The result identified factors including *stimulation method*, *target hemisphere*, *target area*, and *stimulation timing*. Each moderating factor was separately discussed below.

#### Stimulation method

Our result indicated that the effect size of TMS studies was significantly larger than that of anodal tDCS studies, which aligns with the results of previous study [43]. This finding may be attributed to differences in the electric field and focality of the two techniques. Evidence from imaging and computational modeling have revealed that in comparison to TMS, tDCS is vulnerable to anatomical factors, such as the thickness of skull and cerebrospinal fluid, which may lead to up to 50% of the electric field intensity being affected [94–96]. Moreover, in terms of focal stimulation, TMS exhibits higher spatial precision than tDCS, resulting in a more focused stimulation of the target area [22, 26]. Therefore, compared to tDCS, TMS is a more efficient and promising tool to improve down-regulation function.

#### Target area and target hemisphere

Our result indicated that the effect size of targeting the rVLPFC is significantly greater than that of lDLPFC, lVLPFC, and rDLPFC. This finding indicated that rVLPFC was the golden target to stimulate to obtain effects on down-regulation. Neuroimage meta-analysis and lesion studies have shown that the VLPFC plays a critical role in down-regulation [14, 15, 97], particularly rVLPFC [98, 99]. Moreover, rVLPFC is also a critical region for inhibition [100, 101]. During down-regulation of emotion, rVLPFC involved the inhibition of negative emotion [20, 99, 102]. A recent TMS study provides causal evidence that further supports the inhibitory role of rVLPFC during down-regulation [103]. Therefore, TMS and tDCS targeting the right VLPFC can produce a larger effect on down-regulation. In addition, we also identified the hemispheric asymmetry in the TMS and tDCS effect on down-regulation, with studies targeting right PFC exhibiting significantly larger effect sizes than those targeting the left PFC. One possible explanation for this finding would be that negative emotions are more closely associated with the right PFC [104–106]. In summary, the present findings suggest that right PFC, especially rVLPFC, may be an optimal site for potential intervention in down-regulation.

#### Stimulation timing

Results suggested that offline TMS and tDCS produced larger effect sizes than online TMS and tDCS. However, it should be noted that all included offline studies were TMS studies, and most online studies were tDCS studies. Cautions should be taken as we cannot rule out the possibility that the modulation effect comes from stimulation method because the effect of TMS is better than tDCS (see the result of *stimulation method*).

### Clinical implications

Deficits in ER are recognized as a core feature of various psychiatric conditions, including major depressive disorder [107, 108], anxiety disorders [109], and autism spectrum disorder [110]. The current findings may hold clinical implications for developing targeted neuromodulation treatments for these disorders. The divergent effects of TMS and tDCS on explicit versus implicit ER suggest protocols could be tailored based on the specific regulation impairments exhibited in a given patient population. Numerous studies, for example, have shown that depressed individuals exhibit compromised explicit ER function [3, 111–113], while their implicit ER function remains unaffected. therapeutic approaches for depressed individuals should be tailored to target the neural circuit of explicit ER. It is also notable that a single session of TMS and tDCS in healthy individuals yielded only small effects in this meta-analysis (*Hedges' g* = − 0.20), which is consistent with previous emotion and er meta-analysis studies [42, 43]. Achieving durable clinical improvements requires repeated TMS and tDCS sessions over weeks to induce synaptic plasticity [114, 115]. Rigorously testing the efficacy and safety of such multi-session protocols in clinical populations will be an important direction before translational application.

## Limitations

Several limitations should be put forward. First, although our result demonstrated a positive result on physiological response of down-regulation (3 studies) as well as a null result on implicit ER (5 studies), these findings were not strong enough because of the small number of studies. Further research is required to validate these results. Second, studies included in our meta-analysis mainly used cognitive reappraisal and fear extinction task, only three studies used other tasks [39, 72, 116]. Such high homogeneity makes it difficult to test whether the TMS and tDCS effect varies from different explicit and implicit ER tasks, such as distraction and emotional Stroop task. Therefore, a more diverse explicit and implicit task should be adopted to investigate the TMS and tDCS on ER. Third, besides PFC, other regions including the temporoparietal junction [117], and cerebellum [118] also play an important role in ER. Future meta-analysis could broaden the target area of TMS and tDCS. Fourth, studies included in our meta-analysis primarily utilized the 10–20 EEG system for target localization. While this method is widely accepted, it may present limitations regarding targeting accuracy due to interindividual differences in head shape and cortical anatomy [119, 120]. On the other hand, neuronavigation, which matches MRI-based 3D models of an individual's head and brain images with the actual subject's head [121], offers an individually tailored approach for optimal target localization. While we recognize the potential benefits of neuronavigation, due to its limited usage in the studies we reviewed (only one employed neuronavigation), we could not perform a sub-analysis to determine if this method would yield superior results. Accordingly, we urge future research to explore the use of neuronavigation for more precise localization.

## Conclusion

In conclusion, we provided physiological evidence that excitatory TMS and tDCS promotes down-regulation function and identified distinct TMS and tDCS effects on explicit and implicit ER, i.e., TMS and tDCS promotes only explicit but not implicit ER. This distinction between explicit and implicit ER highlights the importance of developing TMS and tDCS protocols that are tailored to different forms of ER. In addition, our moderation analysis indicates a protocol that may achieve an optimal TMS and tDCS effect on down-regulation, i.e., adopting high-frequency rTMS or targeting the rVLPFC, or stimulating in an offline manner. Findings may help refine the scope and usage of the TMS and tDCS protocol, thereby optimizing the effectiveness of TMS and tDCS on ER function.

### Supplementary Information

**Additional file 1: Table S1.** Characteristics of inhibitory TMS of explicit and implicit ER studies. **Table S2.** Characteristics of cathode tDCS of explicit and implicit ER studies. **Table S3.** The effect of excitatory and inhibitory TMS and tDCS on the no-regulation condition of explicit ER. **Table S4.** The effect of excitatory and inhibitory TMS and tDCS on the no-regulation condition of implicit ER. **Figure S1.** Risk of bias summary of all included studies (n = 6). (A) Methodological quality assessment of each study at 6 domains was illustrated. (B) Risk of bias graph. **Figure S2.** Forest plot for the summary effect size on the effect of inhibitory TMS and tDCS on the subjective experience of down-regulation. Combied: Studies with multiple outcomes within a study were combined into averaged data with CMA, which can prevent an improper estimate of the precision of the summary effect. **Figure S3.** Forest plot for the summary effect size on the effect of inhibitory TMS and tDCS on subjective experience of up-regulation. **Figure S4.** Forest plot for the summary effect size on the effect of inhibitory TMS and tDCS on subjective experience of implicit ER. Combied: Studies with multiple outcomes (e.g. valence and arousal) within a study were combined into averaged data with CMA, which can prevent an improper estimate of the precision of the summary effect. **Figure S5.** Sensitivity analysis with one study removed for the excitatory TMS and tDCS effect on the subjective experience of down-regulation. **Figure S6.** Sensitivity analysis with one study removed for the excitatory TMS and tDCS effect on the physiological response of down-regulation. **Figure S7.** Sensitivity analysis with one study removed for the excitatory TMS and tDCS effect on the subjective experience of up-regulation. **Figure S8.** Sensitivity analysis with one study removed for the excitatory TMS and tDCS effect on the physiological response of implicit ER.

## Data Availability

The data of this study would be available upon reasonable request and with the approval of the corresponding author, Prof. D. Zhang (zhangdd05@gmail.com).

## Abbreviations

ER: Emotion regulation
PFC: The prefrontal cortex
DLPFC: Dorsolateral prefrontal cortex
VLPFC: Ventrolateral prefrontal cortex
MPFC: Medial prefrontal cortex
VMPFC: Ventral medial prefrontal cortex
tES: Transcranial electrical stimulation
tDCS: Transcranial direct current stimulation
tACS: Transcranial alternating current stimulation
tRNS: Transcranial random noise stimulation
TMS: Transcranial magnetic stimulation
rTMS: Repetitive transcranial magnetic stimulation
spTMS: Single pulse TMS
cTBS: Continuous theta burst stimulation
iTBS: Intermittent theta burst stimulation
PRISMA: Preferred reporting items for systematic reviews and meta-analyses
CI: Confident interval
