# Supplementary material for: Transcranial magnetic stimulation and transcranial direct current stimulation affect explicit but not implicit emotion regulation: a meta-analysis
Source: Behav Brain Funct. 2023 Sep 19;19:15. doi: 10.1186/s12993-023-00217-8 (PMC10510188; doi:10.1186/s12993-023-00217-8)
Supplement: Supplementary file 1 — Additional file 1: Table S1. Characteristics of inhibitory TMS of explicit and implicit ER studies. Table S2. Characteristics of cathode tDCS of explicit and implicit ER studies. Table S3. The effect of excitatory and inhibitory TMS and tDCS on the no-regulation condition of explicit ER. Table S4. The effect of excitatory and inhibitory TMS and tDCS on the no-regulation condition of implicit ER. Figure S1. Risk of bias summary of all included studies (n = 6). (A) Methodological quality assessment of each study at 6 domains was illustrated. (B) Risk of bias graph. Figure S2. Forest plot for the summary effect size on the effect of inhibitory TMS and tDCS on the subjective experience of down-regulation. Combied: Studies with multiple outcomes within a study were combined into averaged data with CMA, which can prevent an improper estimate of the precision of the summary effect. Figure S3. Forest plot for the summary effect size on the effect of inhibitory TMS and tDCS on subjective experience of up-regulation. Figure S4. Forest plot for the summary effect size on the effect of inhibitory TMS and tDCS on subjective experience of implicit ER. Combied: Studies with multiple outcomes (e.g. valence and arousal) within a study were combined into averaged data with CMA, which can prevent an improper estimate of the precision of the summary effect. Figure S5. Sensitivity analysis with one study removed for the excitatory TMS and tDCS effect on the subjective experience of down-regulation. Figure S6. Sensitivity analysis with one study removed for the excitatory TMS and tDCS effect on the physiological response of down-regulation. Figure S7. Sensitivity analysis with one study removed for the excitatory TMS and tDCS effect on the subjective experience of up-regulation. Figure S8. Sensitivity analysis with one study removed for the excitatory TMS and tDCS effect on the physiological response of implicit ER. [file 12993_2023_217_MOESM1_ESM.docx]

**Supplementary Materials**

# Part 1 Search terms

(“neuromodulation” OR “neurostimulation” OR “noninvasive brain stimulation” OR “noninvasive cortical stimulation” OR “transcranial brain stimulation” OR “transcranial magnetic stimulation” OR “theta burst stimulation” OR “transcranial alternating current stimulation” OR “transcranial electrical stimulation” OR “transcranial direct current stimulation” OR “transcranial random noise stimulation”) AND (“affect labeling” OR “affect regulation” OR “automatic goal pursuit” OR “distancing” OR “downregulation” OR “emotion regulation” OR “emotional Go/No-Go” OR “emotional regulation” OR “emotional Stroop” OR “extinction” OR “placebo” OR “reappraisal” OR “reinforcer revaluation” OR “reinterpretation” OR “reversal learning” OR “selective attention” OR “suppression” OR “upregulation”)

# Part 2 The effect of inhibitory TMS and tDCS on ER

## Methods

We also calculated effect size (*Hedges' g*) to assess the effect of inhibitory TMS and tDCS on ER and no-regulation condition. Negative values indicate decreased, while positive values indicate increased negative emotional reactivity in active condition compared to sham condition. The methods for data extraction and analysis, as well as the assessment of risk of bias, were the same as those used in the main text.

## Results

### Literature search and study characteristics

A total of 6 studies (explicit ER: n = 4; implicit ER: n = 2) were included. These studies including low-frequency rTMS, inhibitory spTMS, and cathode tDCS targeted over DLPFC and VLPFC. For detailed information on study characteristics see Tables S1 and S2.

### Risk of bias

A summary of risk of bias assessment of inhibitory TMS and tDCS studies was illustrated in Figure S1. The result of the risk of bias showed that 3 (50%) studies had a low risk of bias, 1 (16.7%) study was an unclear risk, and 2 (33.3 %) studies were of high risk of bias. The problem of missing outcomes data and the randomization process was responsible for the unclear and high risk.

### The effect of inhibitory TMS and tDCS on explicit ER

*Subjective experience of* *down-regulation*

Four studies were included. Two studies targeting rVLPFC or rDLPFC did find an inhibitory TMS and tDCS effect on negative emotion during down-regulation [1, 2]. But, the other two studies stimulating lVLPFC or lDLPFC did not demonstrate such an effect [3, 4].

This analysis included 183 participants, of which 31 underwent both active and sham stimulation, 77 only underwent active stimulation, and 75 only sham stimulation. The full random effects model showed no significant inhibitory stimulation effect on subjective experience of down-regulation (*Hedges' g* = 0.39, *Z*-value = 1.26; 95% CI = [-0.22, 1.00]; *p* = 0.208) (Figure S2), indicating that compared to sham stimulation, inhibitory stimulation over PFC could not influence the negative emotional experience during down-regulation. High heterogeneity was observed (*Q* = 11.75; *p* = 0.008; *I^2^* = 74.46%). For smaller samples, subgroup analysis could not perform.

*Subjective experience of up-regulation*

We identified two studies examining the effect of inhibitory stimulation on the subjective experience of up-regulation [2, 4], which found no effect.

This analysis included 79 participants, of which 41 only underwent active stimulation and 38 only sham stimulation. The full random effects model showed no significant inhibitory stimulation effect on subjective experience of up-regulation (*Hedges' g* = -0.05, *Z*-value = -0.20; 95% CI = [-0.48, 0.39]; *p* = 0. 0.838) (Figure S3). Low heterogeneity was observed (*Q* = 0.05; *p* = 0.827; *I^2^* = 0.00%). For smaller samples, subgroup analysis could not perform.

*Physiological response of down-regulation*

We did not identify studies examining the effect of inhibitory stimulation on the physiological response of down-regulation.

*Physiological response of up-regulation*

We did not identify studies examining the effect of inhibitory stimulation on the physiological response of up-regulation.

### The effect of inhibitory TMS and tDCS on implicit ER

*Subjective experience*

Two studies were included which did not find an inhibitory effect on valence and arousal of negative stimulus [5, 6].

This analysis included 55 participants, of which 12 underwent both active and sham stimulation, 27 only underwent active stimulation and 16 only sham stimulation. No significant inhibitory stimulation effect on subjective experience of implicit ER was found (*Hedges' g* = -0.06, *Z-*value = -0.23; 95% CI = [-0.53, 0.42]; p = 0.818) (Figure S4). Low heterogeneity was observed (*Q* = 0.13; *p* = 0.720; *I^2^* = 0.00%). Because of the limited sample size, moderation analysis could not perform.

*Physiological response*

One study was included but found no effect of inhibitory stimulation on physiological arousal of implicit ER [5].

### The effect of inhibitory TMS and tDCS on the no-regulation condition

We also calculated the inhibitory TMS and tDCS effect on the no-regulation condition of explicit and implicit ER. The result showed that there was no significant TMS and tDCS effect on the self-reported and physiological results (*p* > 0.05) (see Table S3 and Table S4 in part 3).

## Discussion

We investigated the effect of inhibitory TMS and tDCS on ER. We did not find evidence that inhibitory TMS and tDCS affects both explicit and implicit ER. It is noteworthy that the two studies targeting the rVLPFC and rDLPFC found inhibitory effects of TMS and tDCS on down-regulation [1, 2], but there were no results when targeting the left part [3, 4]. These results suggest that the inhibition of TMS and tDCS may have hemispherical asymmetry, which is consistent with the results of excitatory TMS and tDCS on down-regulation. However, due to the limitation of the number of studies, further analysis could not be performed.

Overall, the limited sample size may contribute to these null effects, thus more research is needed to explore the effect of inhibitory TMS and tDCS on ER.

## References

1. Cheng S, Qiu X, Li S, Mo L, Xu F, Zhang D. Different Roles of the Left and Right Ventrolateral Prefrontal Cortex in Cognitive Reappraisal: An Online Transcranial Magnetic Stimulation Study. Frontiers in Human Neuroscience. 2022;16. https://doi.org/10.3389/fnhum.2022.928077.

2. Tu Y, Wilson G, Camprodon J, Dougherty DD, Vangel M, Benedetti F, et al. Manipulating placebo analgesia and nocebo hyperalgesia by changing brain excitability. Proceedings of the National Academy of Sciences. 2021;118(19):e2101273118. https://doi.org/doi:10.1073/pnas.2101273118.

3. de Wit SJ, van der Werf YD, Mataix-Cols D, Trujillo JP, van Oppen P, Veltman DJ, et al. Emotion regulation before and after transcranial magnetic stimulation in obsessive compulsive disorder. Psychol Med. 2015;45(14):3059-73. https://doi.org/10.1017/s0033291715001026.

4. Vieira L, Marques D, Melo L, Marques RC, Monte-Silva K, Cantilino A. Transcranial direct current stimulation effects on cognitive reappraisal: An unexpected result? Brain Stimulation. 2020;13(3):650-2. https://doi.org/10.1016/j.brs.2020.02.010.

5. Ganho-Ávila A, Gonçalves Ó F, Guiomar R, Boggio PS, Asthana MK, Krypotos AM, et al. The effect of cathodal tDCS on fear extinction: A cross-measures study. PloS One. 2019;14(9):e0221282. https://doi.org/10.1371/journal.pone.0221282.

6. Urgesi C, Mattiassi AD, Buiatti T, Marini A. Tell it to a child! A brain stimulation study of the role of left inferior frontal gyrus in emotion regulation during storytelling. Neuroimage. 2016;136:26-36. https://doi.org/10.1016/j.neuroimage.2016.05.039.

## Figures and tables

Table S1. Characteristics of inhibitory TMS of explicit and implicit ER studies.

| Author | Design Sample size n(active)\|n(control) | Coil position  (localization method) | Stimulation frequency,  Quantity, Intensity | Control condition | Timing | Stimuli type | Task types | ER goals | Measurement and result |
| --- | --- | --- | --- | --- | --- | --- | --- | --- | --- |
| ***explicit ER* (n = 2, k = 3)** | | | | | | | | | |
| **spTMS** | | | | | | | | | |
| Cheng et al., 2022(I) | within-subjects  31\|31^a^ | lVLPFC (F7, 10-20) | spTMS, 1 pulse,  120% rMT | Cz, 10–20 | online | social exclusion pictures | ERT | down | negative feeling: active = sham |
| Cheng et al., 2022(II) | within-subjects  31\|31^a^ | rVLPFC (F8, 10-20) | spTMS, 1 pulse,  120% rMT | Cz, 10–20 | online | social exclusion pictures | ERT | down | negative feeling: active > sham |
| **low-frequency rTMS** | | | | | | | | | |
| De Wit et al., 2015; | between-subjects 19\|19 | lDLPFC (neuronavigation) | 1 Hz，1200 pulses， 110% rMT | Cz, 10–20 | offline | fearful pictures | ERT | down | distress: active = sham |
| ***implicit ER* (n = 1, k = 1)** | |  |  |  |  |  |  |  |  |
| **cTBS** |  |  |  |  |  |  |  |  |  |
| Urgesi et al., 2016 | within-subjects 12\|12 | lVLPFC (neuronavigation) | 50 Hz, 600 pulses,  80% rMT | Cz, 10–20 | offline | negative IAPS pictures | ERT | down | valence: active = sham |

n is the number of studies; k is the number of outcomes; ^a^Samples used for multiple experiments within a study.

ER = emotion regulation; ERT = emotion regulation task; down = down-regulation; l = left; r = right; VLPFC = ventrolateral prefrontal cortex; DLPFC = dorsolateral prefrontal cortex; spTMS = single pulse transcranial magnetic; low-frequency rTMS = low-frequency repetitive transcranial magnetic stimulation; cTBS = continuous theta burst stimulation; rMT = resting motor threshold; 10–20 = 10–20 system for localizing scalp electrodes; IAPS = International Affective Picture System.

Table S2. Characteristics of cathode tDCS of explicit and implicit ER studies.

| Author | Design Sample size n(active)\|n(control) | Electrode positions  (localization method) | Current intensity,  anode + cathode size, quantity | Control condition (time of current ramped down) | Timing | Stimuli type | Task types | ER goal | Measurement and result |
| --- | --- | --- | --- | --- | --- | --- | --- | --- | --- |
| ***explicit ER* (n = 2, k = 2)** | | | | | | | | | |
| Vieira et al., 2020(II) | between-subjects 14\|11 ^a^ | lVLPFC (anode, F7;  cathode, Fp2, 10–20) | 1 mA,  9 + 25 cm², 20 min | 30 s | online | negative pictures | ERT | down  up | arousal: active = sham arousal: active = sham |
| Tu et al., 2021 | between-subjects  27\|27 | rDLPFC (cathodal, F4; anodal, FP1, 10–20) | 2 mA,  16 + 16 cm²,  20 min | 15 s | online | heat stimuli | PNT | down | pain rating: active > sham |
|  |  |  |  |  |  |  |  | up | pain rating: active = sham |
| ***implicit ER* (n = 1, k = 1)** | |  |  |  |  |  |  |  |  |
| Ganho-Ávila et al., 2019; | between-subjects (females only) 27\|16 | rDLPFC (cathode, F4;  anode, contralateral deltoid, 10–20) | 1 mA,  24.75 + 24.75 cm²,  20 min | 30 s | offline | 95-db aversive screams | FET | down | valence, arousal: active = sham SCR: active = sham |

n is the number of studies; k is the number of outcomes; ^a^Samples used for multiple experiments within a study.

ER = emotion regulation; ERT = emotion regulation task; FET = fear extinction task; PNT = placebo nocebo Task; down = down-regulation; up = up-regulation; l = left; r = right; VLPFC = ventrolateral prefrontal cortex; DLPFC = dorsolateral prefrontal cortex; ctDCS = cathode transcranial direct current stimulation; 10–20 = 10–20 system for localizing scalp electrodes; IAPS = International Affective Picture System; SCR = skin conductance response.


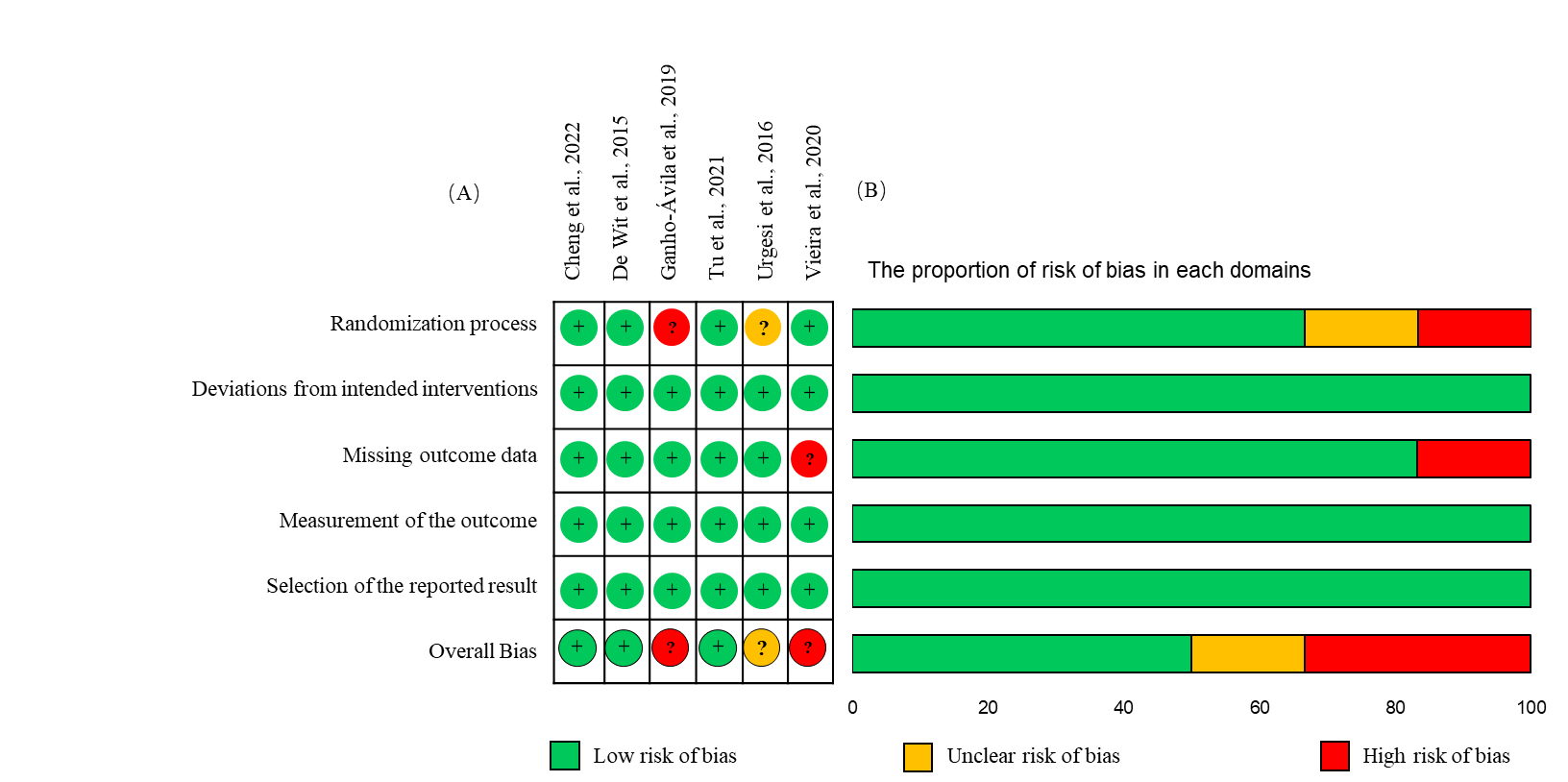


Figure S1. Risk of bias summary of all included studies (n = 6). (A) Methodological quality assessment of each study at 6 domains was illustrated. (B) Risk of bias graph.


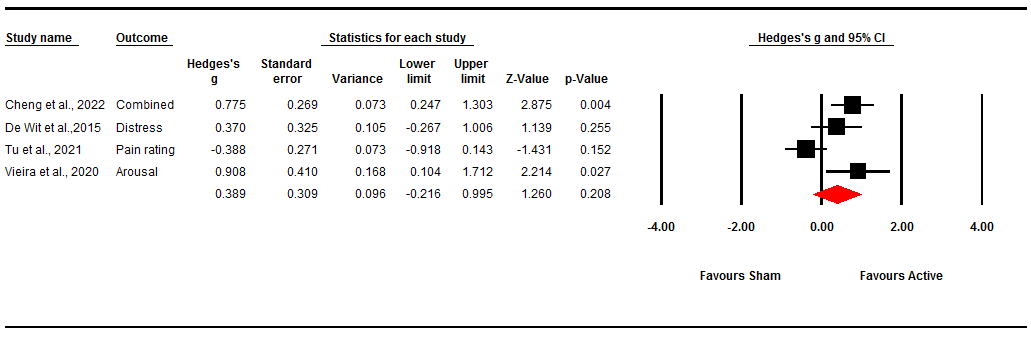


Figure S2. Forest plot for the summary effect size on the effect of inhibitory TMS and tDCS on the subjective experience of down-regulation. Combied: Studies with multiple outcomes within a study were combined into averaged data with CMA, which can prevent an improper estimate of the precision of the summary effect.


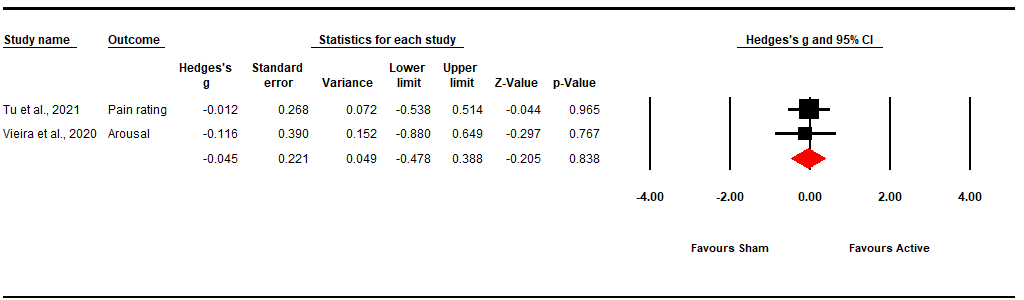


Figure S3. Forest plot for the summary effect size on the effect of inhibitory TMS and tDCS on subjective experience of up-regulation.


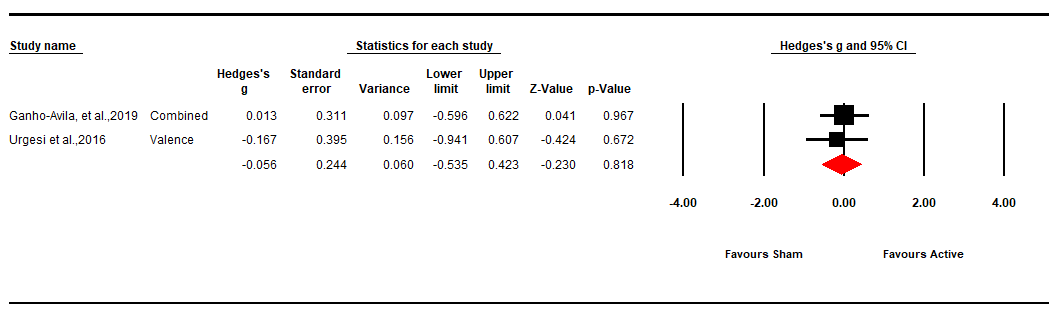


Figure S4. Forest plot for the summary effect size on the effect of inhibitory TMS and tDCS on subjective experience of implicit ER. Combied: Studies with multiple outcomes (e.g. valence and arousal) within a study were combined into averaged data with CMA, which can prevent an improper estimate of the precision of the summary effect.

# Part 3: The effect of TMS and tDCS on the no-regulation condition of explicit and implicit ER

Table S3. The effect of excitatory and inhibitory TMS and tDCS on the no-regulation condition of explicit ER

|  | *k* | *Hedges' g,* (95% CI) | *Z* | *p^a^* | *Q* | *df* | *p^b^* | *I^2^(%)* |
| --- | --- | --- | --- | --- | --- | --- | --- | --- |
| **Excitatory TMS and tDCS** | |  |  |  |  |  |  |  |
| *Subjective experience* | | | | | | | | |
| down-regulation | 24 | -0.12, (-0.26, -0.02) | -1.62 | 0.105 | 36.41 | 23 | 0.037 | 36.84 |
| up-regulation | 7 | -0.12, (-0.36, 0.13) | -0.93 | 0.351 | 7.76 | 6 | 0.256 | 22.66 |
| *Physiological response* | |  |  |  |  |  |  |  |
| down-regulation | 3 | -0.41, (-0.67, -0.15) | -3.09 | *P* < 0.001 | 0.07 | 2 | 0.964 | 0.00 |
| up-regulation | 1 | / | / | / | / | / | / | / |
| **Inhibitory TMS and tDCS** | |  |  |  |  |  |  |  |
| *Subjective experience* | | | | | | | | |
| down-regulation | 4 | 0.43, (-0.36, 1.23) | 1.07 | 0.286 | 21.04 | 3 | *P* < 0.001 | 85.74 |
| up-regulation | 2 | 0.00, (-0.43, 0.43) | 0.00 | 1.000 | 0.13 | 1 | 0.719 | 0.00 |
| *Physiological response* | |  |  |  |  |  |  |  |
| down-regulation | / | / | / | / | / | / | / | / |
| up-regulation | / | / | / | / | / | / | / | / |

Abbreviations: CI = confidence interval; *df* = degree of freedom; *Q* = Cochran’s Q, assess the presence of heterogeneity; *I^2^* = assess the magnitude of the heterogeneity; *p*^a^ = the p value for effect size (*Hedges' g*); *p*^b^ = the *p* value for heterogeneity test (*Cochran’s Q*).

Table S4. The effect of excitatory and inhibitory TMS and tDCS on the no-regulation condition of implicit ER

|  |  | *k* | *Hedges' g,* (95% CI) | *Z* | *p^a^* | *Q* | *df* | *p^b^* | *I^2^(%)* |
| --- | --- | --- | --- | --- | --- | --- | --- | --- | --- |
| **Excitatory TMS and tDCS** | | | |  | | | | | |
| *Subjective experience* | | 1 | / | / | / | / | / | / | / |
| *Physiological response* | | 4 | -0.04, (-0.36, 0.28) | -0.25 | 0.802 | 4.43 | 3 | 0.219 | 32.28 |
| **Inhibitory TMS and tDCS** | | | |  | |  |  |  |  |
| *Subjective experience* | | 2 | 0.02, (-0.45, 0.50) | 0.10 | 0.921 | 0.24 | 1.000 | 0.521 | 0.00 |
| *Physiological response* | | 1 | / | / | / | / | / | / | / |

Abbreviations: CI = confidence interval; *df* = degree of freedom; *Q* = Cochran’s Q, assess the presence of heterogeneity; *I^2^* = assess the magnitude of the heterogeneity; *p*^a^ = the p value for effect size (*Hedges' g*); *p*^b^ = the *p* value for heterogeneity test (*Cochran’s Q*).

# Part 4: Sensitivity analysis


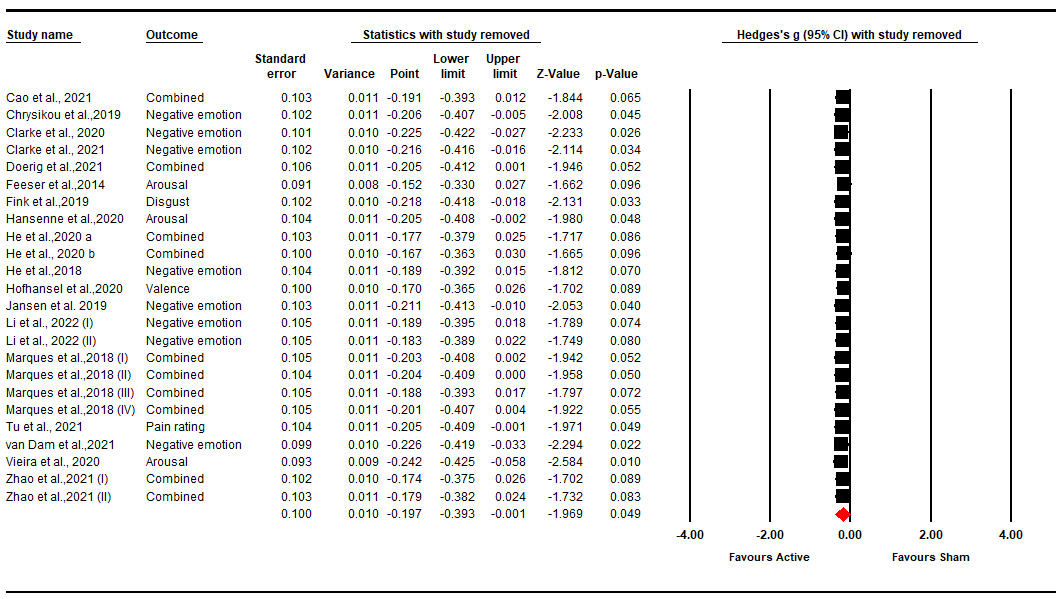


Figure S5. Sensitivity analysis with one study removed for the excitatory TMS and tDCS effect on the subjective experience of down-regulation.


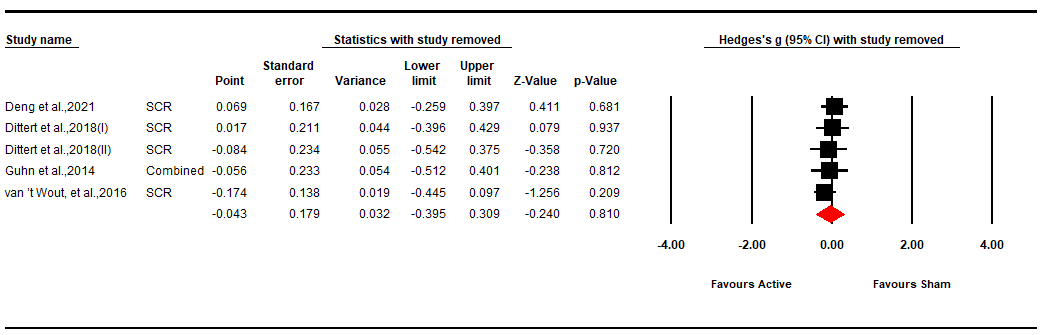


Figure S6. Sensitivity analysis with one study removed for the excitatory TMS and tDCS effect on the physiological response of down-regulation.


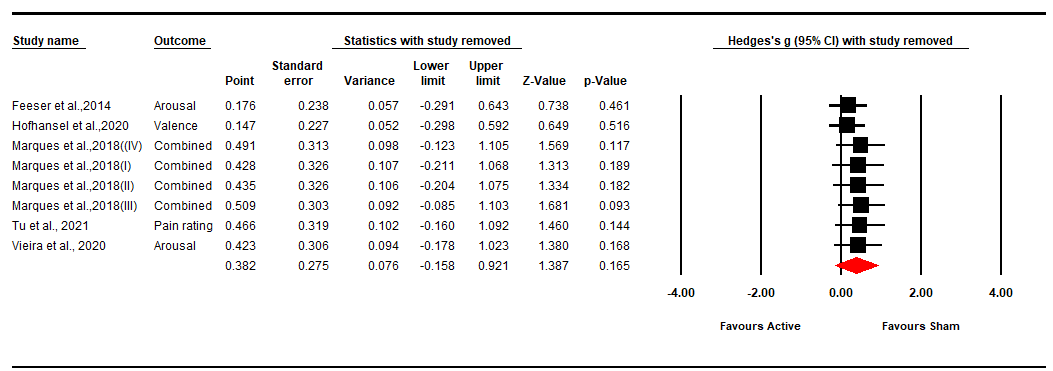


Figure S7. Sensitivity analysis with one study removed for the excitatory TMS and tDCS effect on the subjective experience of up-regulation.


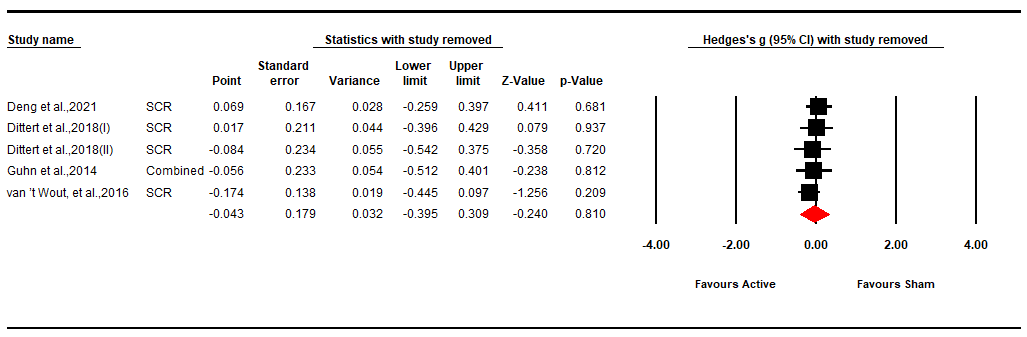


Figure S8. Sensitivity analysis with one study removed for the excitatory TMS and tDCS effect on the physiological response of implicit ER.
